# Safety of single-dose primaquine as a *Plasmodium falciparum* gametocytocide: a systematic review and meta-analysis of individual patient data

**DOI:** 10.1186/s12916-022-02504-z

**Published:** 2022-09-16

**Authors:** Kasia Stepniewska, Elizabeth N. Allen, Georgina S. Humphreys, Eugenie Poirot, Elaine Craig, Kalynn Kennon, Daniel Yilma, Teun Bousema, Philippe J. Guerin, Nicholas J. White, Ric N. Price, Jaishree Raman, Andreas Martensson, Richard O. Mwaiswelo, Germana Bancone, Guido J. H. Bastiaens, Anders Bjorkman, Joelle M. Brown, Umberto D’Alessandro, Alassane A. Dicko, Badria El-Sayed, Salah-Eldin Elzaki, Alice C. Eziefula, Bronner P. Gonçalves, Muzamil Mahdi Abdel Hamid, Akira Kaneko, Simon Kariuki, Wasif Khan, Titus K. Kwambai, Benedikt Ley, Billy E. Ngasala, Francois Nosten, Joseph Okebe, Aaron M. Samuels, Menno R. Smit, Will J. R. Stone, Inge Sutanto, Feiko Ter Kuile, Roger C. Tine, Alfred B. Tiono, Chris J. Drakeley, Roly Gosling, Andy Stergachis, Karen I. Barnes, Ingrid Chen

**Affiliations:** 1 WorldWide Antimalarial Resistance Network, Oxford, UK; 2 Centre for Tropical Medicine and Global Health, Nuffield Department of Clinical Medicine, University of Oxford, Oxford, UK; 3 Division of Clinical Pharmacology, Department of Medicine, University of Cape Town, Cape Town, South Africa; 4 Green Templeton College, University of Oxford, Oxford, UK; 5 Malaria Elimination Initiative, Global Health Group, University of California San Francisco, San Francisco, USA; 6 Jimma University Clinical Trial Unit, Department of Internal Medicine, Jimma University, Jimma, Ethiopia; 7 Department of Infection and Immunity, London School of Hygiene and Tropical Medicine, London, UK; 8 Department of Medical Microbiology, Radboud University Medical Center, Nijmegen, The Netherlands; 9 Faculty of Tropical Medicine, Mahidol University, Bangkok, Thailand; 10 Global and Tropical Health Division, Menzies School of Health Research and Charles Darwin University, Darwin, Australia; 11 Parasitology Reference Laboratory, National Institute for Communicable Diseases, A Division of the National Health Laboratory Services, Johannesburg, South Africa; 12 Wits Research Institute for Malaria, Faculty of Health Sciences, University of Witwatersrand, Johannesburg, South Africa; 13 Department of Women’s and Children’s Health, International Maternal and Child Health (IMCH), Uppsala University, Uppsala, Sweden; 14 Department of Parasitology and Medical Entomology, Muhimbili University of Health and Allied Sciences, Dar es Salaam, Tanzania; 15 Department of Microbiology, Immunology and Parasitology, Hubert Kairuki Memorial University, Dar es Salaam, Tanzania; 16 Shoklo Malaria Research Unit, Mahidol-Oxford Tropical Medicine Research Unit, Faculty of Tropical Medicine, Mahidol University, Mae Sot, Thailand; 17 Laboratory of Medical Microbiology and Immunology, Rijnstate Hospital, Arnhem, The Netherlands; 18 Department of Microbiology Tumor and Cell Biology, Karolinska Institutet, Stockholm, Sweden; 19 Department of Epidemiology and Biostatistics, University of California San Francisco, San Francisco, CA USA; 20 Medical Research Council Unit, London School of Hygiene and Tropical Medicine, Fajara, The Gambia; 21 Malaria Research and Training Centre, Faculty of Pharmacy and Faculty of Medicine and Dentistry, University of Science, Techniques and Technologies of Bamako, Bamako, Mali; 22 Department of Epidemiology, Tropical Medicine Research Institute, National Centre for Research, Khartoum, Sudan; 23 Department of Global Health and Infection, Brighton and Sussex Medical School, University of Sussex, Brighton, UK; 24 Institute of Endemic Diseases, University of Khartoum, Khartoum, Sudan; 25 Kenya Medical Research Institute (KEMRI), Kisian, Kenya; 26 Infectious Disease Division, International Centre for Diarrheal Diseases Research, Dhaka, Bangladesh; 27 Centers for Disease Control and Prevention, Department of Parasitic Diseases and Malaria, Kisumu, Kenya; 28 Department of Women’s and Children’s Health, International Maternal and Child Health (IMCH), Uppsala University, Uppsala, Sweden; 29 Disease Control & Elimination Theme, Medical Research Council Unit, Fajara, The Gambia; 30 Liverpool School of Tropical Medicine, Liverpool, UK; 31 Department of Parasitology, Faculty of Medicine, University of Indonesia, Depok City, Indonesia; 32 Department of Medical Parasitology, Faculty of Medicine, University Cheikh Anta Diop, Dakar, Senegal; 33 Department of Biomedical Sciences, Centre National de Recherche et de Formation sur le Paludisme, Ouagadougou, Burkina Faso; 34 Department of Infection Biology, London School of Tropical Medicine and Hygiene, London, UK; 35 Departments of Pharmacy & Global Health, Schools of Pharmacy and Public Health, University of Washington, Seattle, USA

**Keywords:** Malaria, Primaquine, Clinical trial, Safety, Haemoglobin, Haemoglobinuria adverse events, Individual patient data (IPD), Meta-analysis, Systematic review

## Abstract

**Background:**

In 2012, the World Health Organization (WHO) recommended single low-dose (SLD, 0.25 mg/kg) primaquine to be added as a *Plasmodium (P.) falciparum* gametocytocide to artemisinin-based combination therapy (ACT) without glucose-6-phosphate dehydrogenase (G6PD) testing, to accelerate malaria elimination efforts and avoid the spread of artemisinin resistance. Uptake of this recommendation has been relatively slow primarily due to safety concerns.

**Methods:**

A systematic review and individual patient data (IPD) meta-analysis of single-dose (SD) primaquine studies for *P. falciparum* malaria were performed. Absolute and fractional changes in haemoglobin concentration within a week and adverse effects within 28 days of treatment initiation were characterised and compared between primaquine and no primaquine arms using random intercept models.

**Results:**

Data comprised 20 studies that enrolled 6406 participants, of whom 5129 (80.1%) had received a single target dose of primaquine ranging between 0.0625 and 0.75 mg/kg. There was no effect of primaquine in G6PD-normal participants on haemoglobin concentrations. However, among 194 G6PD-deficient African participants, a 0.25 mg/kg primaquine target dose resulted in an additional 0.53 g/dL (95% CI 0.17–0.89) reduction in haemoglobin concentration by day 7, with a 0.27 (95% CI 0.19–0.34) g/dL haemoglobin drop estimated for every 0.1 mg/kg increase in primaquine dose. Baseline haemoglobin, young age, and hyperparasitaemia were the main determinants of becoming anaemic (Hb < 10 g/dL), with the nadir observed on ACT day 2 or 3, regardless of G6PD status and exposure to primaquine. Time to recovery from anaemia took longer in young children and those with baseline anaemia or hyperparasitaemia. Serious adverse haematological events after primaquine were few (9/3, 113, 0.3%) and transitory. One blood transfusion was reported in the primaquine arms, and there were no primaquine-related deaths. In controlled studies, the proportions with either haematological or any serious adverse event were similar between primaquine and no primaquine arms.

**Conclusions:**

Our results support the WHO recommendation to use 0.25 mg/kg of primaquine as a *P. falciparum* gametocytocide, including in G6PD-deficient individuals. Although primaquine is associated with a transient reduction in haemoglobin levels in G6PD-deficient individuals, haemoglobin levels at clinical presentation are the major determinants of anaemia in these patients.

**Trial registration:**

PROSPERO, CRD42019128185

**Supplementary Information:**

The online version contains supplementary material available at 10.1186/s12916-022-02504-z.

## Background

Primaquine is an 8-aminoquinoline drug that kills the hypnozoites of *Plasmodium (P.) vivax* and *P. ovale* and sterilises the mature *P. falciparum* gametocytes, the parasite stage responsible for the transmission of *P. falciparum* malaria from humans to mosquitoes [1]. The World Health Organization (WHO) initially recommended the addition of a 0.75 mg base/kg primaquine dose to the standard treatment of *P. falciparum* malaria in areas of low malaria transmission [2]. As sterilisation precedes gametocyte clearance, a review of transmission-blocking dynamics (based on mosquito infectivity assessments) in 2012 allowed for the construction of a dose-response relationship. This relationship matrix suggested that a 0.25 mg base/kg dose of primaquine would provide maximum gametocyte sterilisation while posing a minimal haemolytic risk, including in glucose-6-phosphate dehydrogenase (G6PD)-deficient individuals. The dose-response relationship for haemolysis in G6PD deficiency is well characterised [3], predominantly in adult individuals with the African A− variant [4]. Thus, the available pharmacodynamic evidence suggests that a single 0.25 mg/kg dose of primaquine is unlikely to cause serious toxicity in G6PD deficiency variants, but this has not been evaluated at scale in routine clinical practice.

Following an expert panel review of the efficacy and safety data on Single low dose (SLD) primaquine, the WHO recommended that the 0.75mg base/kg single *P. falciparum* transmission-blocking dose of primaquine be replaced by the lower dose of 0.25 mg/kg base without the requirement for prior G6PD testing; countries moving towards the elimination of malaria or needing to counter resistance to artemisinin-based antimalarials were encouraged to add this dose of primaquine to artemisinin-based combination therapies (ACTs) [5]. Pregnant women, infants < 6 months of age, and breastfeeding women with infants < 6 months of age were excluded from this recommendation due to limited evidence on safety and birth outcomes [6].

Implementation of WHO’s SLD primaquine recommendation to block *P. falciparum* transmission has been slow, particularly in Africa, where national malaria control programmes with limited primaquine experience expressed the need for more evidence on the haemolytic risks, particularly among individuals with G6PD deficiency. This safety profile is especially important for non-curative drugs that are meant to benefit the community and not necessarily the dosed individual [7]. SLD primaquine deployment is further challenged by the need to crush and dissolve the primaquine table in water prior to administration in children according to a weight-based dosing schedule [8, 9]. Malaria control programmes concluded it would be easier to administer primaquine in weight- or age-based bands, requiring the establishment of the therapeutic range of SLD primaquine as a *P falciparum* transmission-blocking agent, spanning the lowest efficacious dose to the highest tolerable dose around the 0.25 mg/kg target dose [8, 10]. We conducted two Individula patinet data (IPD) meta-analyses to establish this therapeutic dose range for Single dose (SD) primaquine. The first meta-analysis was on primaquine efficacy, which concluded that maximum transmission blocking could be provided at 0.25 mg/kg, even though higher doses were associated with faster gametocyte clearance, especially when co-administered with dihydroartemisinin-piperaquine [11]. This study is the second meta-analysis and aims to establish the safety of SD primaquine, to inform uptake of the WHO recommendation to use a 0.25 mg/kg dose, and to potentially set a guideline for the implementation of weight- or age-based dosing bands.

## Results

**Fig. 1:**
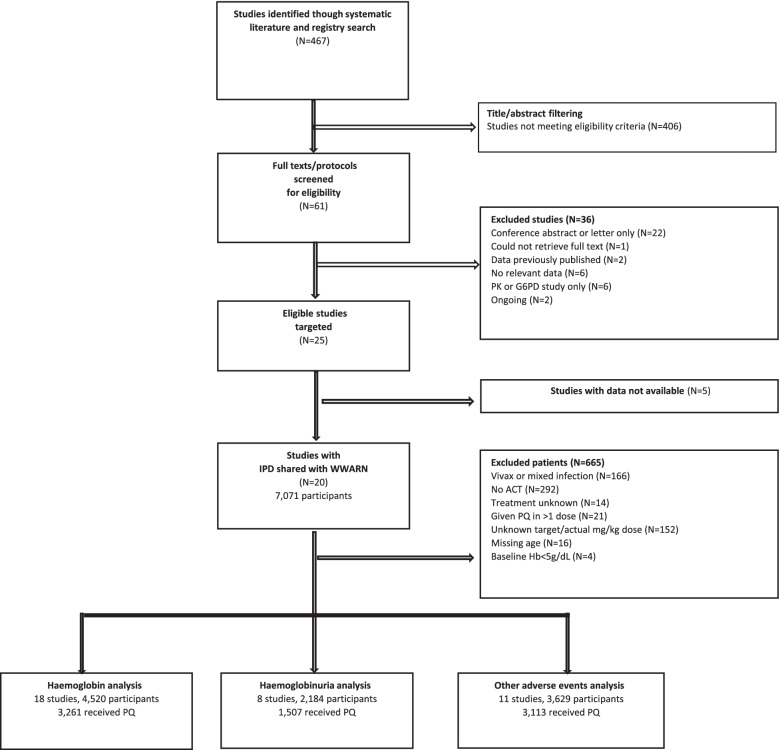
Study profile. Analysis datasets are not mutually exclusive

Literature searches identified 25 eligible studies, for which IPD were available for 20 studies (study IDs 1–20, Additional file 2: List L1): 18 were suitable for the haematology analysis, 11 for the AE analysis, and eight for the haemoglobinuria analysis (Fig. 1). IPD from five eligible studies were not included as data were not available before databases were closed or essential information was missing. Overall, IPD from 6406 participants, 5129 (80.1%) of whom received primaquine, were included in at least one of the analyses.

### Study characteristics

Studies included in this analysis (Table 1, Additional file 2: Table S1) were conducted in 13 countries (10 in Africa, 4181 participants; three in Asia, 2225 participants; Additional file 3: Fig. S1). Fifteen were randomised controlled trials (RCTs) (3305; 51.6% participants), one of which also included cohort data (study ID 9), and five were observational studies comprising two cohort studies (118; 1.8% participants) and three mass drug administration (MDA) studies (2868; 44.8% participants).

**Table 1:** S﻿ummary of the characteristics of the included studies

PMID	Study design	Location	ACT	FU (days)	*N* total	% PQ	Target dose	Age
**1** Unpublished	MDA	Kenya	AP	7	291	98	0.144, 0.208	All
**2 ** 27825738	RCT	The Gambia	DP	42	689	75	0.2, 0.4, 0.75	> 1 year
**3** 27287612	RCT	Tanzania	AL	28	220	50	0.25	≥ 1 year
**4** 29548285	RCT	Sudan	ASSP	42	231	52	0.25	≥ 1 year
**5** 26952094	RCT	Burkina Faso	AL	14	360	69	0.25, 0.4	2–15 years
**6** 18074034	RCT	Sudan	ASSP	14	100	49	0.75	≥ 6 months
**7** Unpublished	RCT	Kenya	DP	42	54	78	0.125, 0.25, 0.4, 0.75	1 ≤ 12 years
**8** 24239324	RCT	Uganda	AL	28	454	75	0.1 0.4 0.75	1–10 years
**9 ** 29324864	RCT and cohort	Burkina Faso	AL	28	78	87	0.25, 0.4	18–45 years
**10** 29324864	RCT	The Gambia	DP	28	61	82	0.25, 0.4	≥ 10 years
**11** 28605472	RCT	Senegal	AL, ASAQ, DP	28	267	50	0.25	> 18 years
**12** 27450652	Cohort	Eswatini	AL	7	94	100	0.25	> 1 year
**13** 17925871	RCT	Tanzania	ASSP	42	107	50	0.75	3–15 years
**14** 20194698	MDA	Tanzania	ASSP	7	840	93	0.75	1–12 years
**15** 23175563	RCT	Indonesia	DP	42	373	52	0.75	≥ 5 years
**16** 31234865	RCT	South Africa	AL	42	140	50	0.25	> 1 year
**17** 28931236	RCT	Kenya	DP	14	114	51	0.25	5–15 years
**18** 27128675	Cohort	Bangladesh	AL	28	115	100	0.75	> 1 year
**19** 26906747	RCT	Mali	DP	28	81	80	0.0625, 0.125, 0.25, 0.5	5–50 years
**20** 27010542	MDA	Thailand	DP	7	1737	100	0.25	> 6 months

*ACT* artemisinin-based combination therapy, *MDA* mass drug administration, *AL* artemether-lumefantrine, *AP* artesunate-piperaquine, *ASAQ* artesunate-amodiaquine, *ASSP* artesunate sulfadoxine-pyrimethamine, *DP* dihydroartemisinin-piperaquine, *PQ* primaquine, *RCT* randomised controlled trial,* FU* follow-up time

The primaquine dose was administered on day 0 (8 studies: 1146; 17.9% participants), day 1 (1 study: 1737; 27.2% participants), day 2 (8 studies: 2910; 45.4% participants) or day 3 (3 studies: 613; 9.5% participants) after the first dose of ACT. Among the 5129 participants treated with primaquine, 49.5% (2540) received the WHO recommended target dose of 0.25 mg/kg, with the previously recommended 0.75 mg/kg target dose given in 29.0% (1487); 9.2% (472) of participants received the 0.40 mg/kg dose; and 0.3% (17) received a 0.50 mg/kg dose. Target primaquine doses below 0.25 mg/kg (0.0625, 0.1, 0.125, 0.144, 0.2, 0.208) were administered in 12.0% (613). The duration of participant follow-up ranged from 7 to 42 days (4 studies day 7, 3 studies day 14, 7 studies day 28, and 6 studies day 42).

The majority of G6PD testing was done using fluorescence spot test (7 studies) or Carestart™ rapid diagnostic tests (RDT) (7 studies). Two studies used PCR (hemi/homozygotes coded as G6PD-deficient), one study used a colourimetric research kit, and one used OSMMR-D-D colourimetric method for quantitative determination. Six studies excluded participants with G6PD deficiency at screening as per their study protocol.

All studies included in the haemoglobin concentration analysis used Hemocue® to measure haemoglobin levels. There were variations in how haemolysis was defined (where such meta-data were available), and none of the included studies measured methaemoglobin concentrations. Two studies monitored haemoglobinuria with direct questioning about dark urine, three with this direct questioning and confirmation with either urine dipstick (two studies) or Hillman test (one study), one study used urine dipsticks only and two Hillman tests only. Systematic enquiries and/or open-ended questions were used to collect health information pertaining to the participant-reported AEs, although the level of detail in these enquiries varied (Additional file 4: Table S13).

### Baseline characteristics of study participants

Most of the 6406 participants were from low-malaria transmission intensity areas (4867, 76.0%). The median age was 10 years (range 6 months to 91 years), with 18.7% (1198) aged below 5 years. At enrolment, participant characteristics were similar between the primaquine and no primaquine groups (Table 2), except for the presence of fever (14.0%, 523/3732 primaquine compared to 39.6%, 404/1021 no primaquine) and severe anaemia (0.01%, 1/3381 primaquine compared to 2.7%, 34/1259 no primaquine). The prevalence of severe anaemia was very low because all studies had an exclusion criterion of haemoglobin at least below 7 g/dL. The difference in baseline haemoglobin levels and presence of fever were largely driven by varied study design and inclusion criteria (Additional file 2: Table S1), reflecting the fact that 80.1% (1026/1281) of participants in the no primaquine group had detectable malaria, compared to only 35.5% (1820/5129) in the primaquine group. Of the 44.4% (2846/6406) of participants with microscopic malaria parasitaemia at baseline, 5.6% (147/2650) had > 100,000 parasites per microliter.

**Table 2:** Baseline ﻿characteristics of patients included in the analysis

	Primaquine		No primaquine		All	
	***N***	***n*** [%] or median [range]	***N***	***n*** [%] or median [range]	***N***	***n*** [%] or median [range]
Sex: male	5127	2912 [56.8]	1277	723 [56.6]	6404	3638 [56.8]
Age	5129	10.0 [0.5–91.0]	1277	11.0 [1.0–84.0]	6406	10.0 [0.5–91.0]
Age category						
< 5 years	5129	985 [19.2]	1277	213 [16.7]	6406	1198 [18.7]
5–11 years	5129	1865 [36.4]	1277	429 [33.6]	6406	2294 [35.8]
12+ years	5129	2279 [44.4]	1277	635 [49.7]	6406	2914 [45.5]
WAZ score^a^	663	− 0.5 [− 5.8–4.0]	163	− 0.6 [− 3.8–2.5]	826	− 0.5 [− 5.8–4.0]
Underweight^a^	663	74 [11.2]	163	23 [14.1]	826	97 [11.7]
Pf Malaria status^b^						
No	5129	1477 [28.8]	1277	175 [13.7]	6406	1652 [25.8]
Yes	5129	1820 [35.5]	1277	1026 [80.3]	6406	2846 [44.4]
Unknown	5129	1832 [35.7]	1277	76 [6.0]	6406	1908 [29.8]
G6PD status: deficient	4442	309 [7.0]	1099	75 [6.8]	5541	384 [6.9]
Pf parasite count (× 10^3^/μL)	1672	6.3 [0.012–1292]	978	6.7 [0.009–432]	2650	6.5 [0.009–1292]
Hyperparasiteamia^c^	1672	112 [6.7]	978	35 [3.6]	2650	147 [5.6]
Temperature (°C)	3731	36.9 [34.1–40.3]	1020	37.0 [34.0–40.5]	4751	36.9 [34.0–40.5]
Fever^d^	3732	523 [14.0]	1021	404 [39.6]	4753	927 [19.5]
Hb (g/dL)	3381	11.9 [6.0–20.8]	1259	11.9 [5.1–18.4]	4640	11.9 [5.1–20.8]
Anaemia^e^						
No	3381	2938 [86.9]	1259	1052 [83.6]	4640	3990 [86.0]
Severe	3381	1 [0.0]	1259	34 [2.7]	4640	35 [0.8]
Moderate	3381	442 [13.1]	1259	173 [13.7]	4640	615 [13.3]
Transmission intensity^f^						
Low	5129	3970 [77.4]	1277	897 [70.2]	6406	4867 [76.0]
Moderate	5129	665 [13.0]	1277	205 [16.1]	6406	870 [13.6]
High	5129	494 [9.6]	1277	175 [13.7]	6406	669 [10.4]
Region						
Africa	5129	3083 [60.1]	1277	1098 [86.0]	6406	4181 [65.3]
Asia	5129	2046 [39.9]	1277	179 [14.0]	6406	2225 [34.7]
ACT						
AL	5129	1094 [21.3]	1277	469 [36.7]	6406	1563 [24.4]
AP	5129	286 [5.6]	1277	5 [0.4]	6406	291 [4.5]
ASAQ	5129	45 [0.9]	1277	47 [3.7]	6406	92 [1.4]
ASSP	5129	1006 [19.6]	1277	272 [21.3]	6406	1278 [20.0]
DP	5129	2698 [52.6]	1277	484 [37.9]	6406	3182 [49.7]
Primaquine dose (mg/kg)	5129	0.3 [0.05–1.9]				
Primaquine target dose (mg/kg)	5129	613 [12.0]				
< 0.25						
0.25	5129	2540 [49.5]				
> 0.25	5129	1976 [38.5]				
Day of primaquine administration based on ACT regimen						
0	5129	848 [16.5]				
1	5129	1737 [33.9]				
2	5129	2231 [43.5]				
3	5129	313 [6.1]				

*ACT* artemisinin combination therapy, *AL* artemether-lumefnantrine, *AP* artemisinin-piperaquine, *ASAQ*artesunate-amodiaquine, *ASSP* artesunate-sulfadoxine-pyrimethamine, *DP* dihydroartemisinin-piperaquine. *Pf Plasmodium falciparum*, *PQ* primaquine

^a^Evaluated in children < 5 years of age, WAZ weight-for-age z-score, underweight is defined as WAZ score < − 2

^b^Non-detectable malaria includes community participants in the MDA studies (*n* = 1017) and study participants who were positive for malaria or had detectable gametocytes at screening (*n* = 635), and unknown malaria includes not tested community participants from MDA studies (*n* = 1848) and study participants who were positive for malaria or had detectable gametocytes at screening (*n* = 60)

^c^Defined as parasitaemia < 100,000/μL

^d^Defined as temperature > 37.5 °C or history of fever

^e^Severe anaemia defined as Hb < 7 g/dL, moderate anaemia defined as 7 g/dL < Hb < 10 g/dL

^f^ Transmission intensity defined based on estimates of *P. falciparum* prevalence rate (PfPR), assuming low transmission for study sites with a PfPR < 0.15, moderate transmission if PfPR 0.15 to < 0.40, and high transmission if PfPR ≥ 0.40

Among participants with no detectable baseline malaria, except for 1017 (61.6%) participants in the MDA studies, all were either positive for malaria (589, 35.7%) or had detectable gametocytes (46, 2.8%) at screening.

Of those with haemoglobin (or haematocrit) recorded at baseline, 13.3% (615/4640) presented with moderate anaemia (Hb 7 to < 10.0 g/dL) and 0.8% (35/4640) had severe anaemia (Hb < 7 g/dL). Fever was present in 19.5% (927/4753) of participants. Of the children < 5 years of age with recorded weight, 11.7% (97/826) were underweight (weight-for-age *z*-score < − 2). G6PD status was classified as deficient in 6.9% (384/5541) tested, and 13.5% (865/6406) of all participants were not tested. Among the 384 participants classified as G6PD-deficient, 42.5% were from Asia, 12.8% were under 5 years of age, 15.6% were females, and 80.5% (309) received primaquine at target doses below 0.25 mg/kg ( 6, 1.9%), equal to 0.25 mg/kg (255, 82.5%), and above 0.25 mg/kg ( 48, 15.5%).

Most of the 6406 participants were treated with either dihydroartemisinin-piperaquine (DP) (3182, 49.7%) or artemether-lumefantrine (AL) (1563, 24.4%). Other ACTs included artesunate-sulfadoxine-pyrimethamine (ASSP) (1282, 20.0%), artemisinin-piperaquine (AP) (291, 4.5%), and artesunate-amodiaquine (ASAQ) (92, 1.4%).

### Analysis of haematological response

Most participants from the 18 studies included in the haematological analysis were from Africa (91.7%, 4147/4520). Their baseline characteristics were similar to the characteristics of the whole dataset (Additional file 3: Table S2) except for a higher number of participants presenting with microscopically detected malaria (60.0%, 2712/4520) and with fever (27.9%, 808/2899).

G6PD deficiency was observed in 221 participants, and 194 of them (127 receiving primaquine) had haemoglobin measurements available on days 0 and 7 and were included in the analysis of day 7 changes. The remaining 27 participants (19 receiving primaquine) had limited haemoglobin information; however, among 12 participants who had at least one haemoglobin measurement after primaquine administration, no follow-up concentrations below 11.1 g/dL were recorded by day 8 (baseline concentrations ranged from 12.8 to 17.3 g/dL).

Haemoglobin data were also available in a further 163 G6PD-deficient participants in two studies from Asia (study ID 18 and 20) which were not included in the haematology analysis set as no day 0 or day 7 haemoglobin data were collected. Haemoglobin concentration did not drop below 8 g/dL in these participants after primaquine administration (Additional file 3: Fig. S2); however, in three participants, the drop between days 1–2 and days 6–9 was > 3 g/dL (3.5, 4.7, 8.2 g/dL drops) but not reported as AEs. This proportion, 2.0% (3/149), compares to 0.8% (12/1523) in G6PD-normal participants in these two studies.

#### Changes in haemoglobin concentrations on day 7

There was no effect of primaquine in G6PD-normal participants on haemoglobin concentrations. However, among the 194 G6PD-deficient individuals (all in Africa), a significant dose-dependent effect was observed on all haematological metrics (Table 3, Additional file 3: Figs. S3 and S4). A 0.25 mg/kg target dose of primaquine in these G6PD-deficient individuals resulted in a median (IQR interquartile range; max value) of 1.3 (0.3–2.2; 6.1) g/dL reduction in haemoglobin concentration (83 participants) compared to 0.7 (− 0.2–1.7; 3.6) g/dL in no primaquine arms (67 participants). Very few G6PD-deficient participants received target primaquine doses above 0.25 mg/kg, with a haemoglobin reduction of 0.8 (0.4–1.2; 3.5) g/dL observed for the 0.40 mg/kg dose (19 participants) and 2.9 (0.4–4.2; 6.5) g/dL for the 0.75 mg/kg dose (19 participants).

**Table 3:** Risk factors for the change in haemoglobin concentration on day 7 following ACT first dose administration

**Parameter**	**Absolute change in haemoglobin concentration**				**Fractional drop > 25% compared to baseline levels**			
	***N***^a^	**change (g/dL)**	**95% CI**	***P*-value**	***n***^a^	**AOR**	**95% CI**	***P*-value**
Age/sex category								
< 5 years	733	− 0.82	− 0.99, − 0.66	< 0.001	27	6.20	2.73, 14.12	< 0.001
5–11 years	1561	− 0.57	− 0.70, − 0.45	< 0.001	35	2.70	1.32, 5.50	0.006
12+ years females	612	− 0.60	− 0.72, − 0.47	< 0.001	28	3.20	1.67, 6.13	< 0.001
12+ males	969	0.00			22	1.00		
Transmission intensity^b^								
Low	2444	− 0.40	− 0.90, 0.10	0.117	96	4.20	1.41, 12.51	0.010
Moderate	769	− 0.38	− 0.62, − 0.14	0.003	11	1.64	0.47, 5.78	0.438
High	662	0.00			5	1.00		
Pf malaria status								
Yes	2462	− 0.72	− 1.20, − 0.24	0.003				
No or unknown^c^	1413	0.00						
Log 10 parasitaemia	3875	Nonlinear^d^		< 0.001				
Haemoglobin (g/dL)	3875	Nonlinear^e^		< 0.001	112		Nonlinear^e^	< 0.001
G6PD status								
Normal	3170	0.00			78	1.00		
Deficient	194	− 0.03	− 0.30, 0.24	0.834	20	1.45	0.51, 4.16	0.486
Unknown	511	− 0.14	− 0.40, 0.13	0.312	14	0.24	0.04, 1.59	0.140
Primaquine dose (0.1 mg/kg)^f^								
Normal G6PD status	2265	− 0.01	− 0.03, 0.01	0.334	55	1.06	0.98, 1.15	0.162
G6PD deficient	127	− 0.27^g^	− 0.34, − 0.19	< 0.001	18	1.63^h^	1.32, 2.01	< 0.001
G6PD status unknown	389	− 0.01	− 0.05, 0.03	0.732	14	1.36	1.04, 1.78	0.026
	**Moderate/severe anaemia (Hb < 10 g/dL)**				**Severe anaemia (Hb < 7 g/dL)**^i^			
	***n***^a^	**AOR**	**95% CI**	***P*-value**	***n***^a^	**AOR**	**95% CI**	***P*-value**
Age/sex category								
< 5 years	294	3.52	2.18, 5.68	< 0.001				
-11 years	234	1.89	1.23, 2.92	0.004				
2+ years: females	68	1.35	0.85, 2.16	0.209				
12+ years: males	43	1.00						
Log 10 parasitaemia (/μL)	639	Nonlinear^d^		< 0.001				
Haemoglobin (g/dL)	639	Nonlinear^e^		< 0.001	35	Nonlinear^e^		< 0.001
G6PD status								
Normal	533	Reference						
Deficient	44	0.99	0.43, 2.24	0.974				
Unknown	62	0.66	0.27, 1.62	0.368				
Primaquine dose (per 0.1 mg/kg increase)^f^								
Normal G6PD status	403	1.04	1.00, 1.09	0.079	9	1.19	0.99, 1.42	0.059
G6PD deficient	33	1.60^k^	1.28, 1.99	< 0.001	6	1.89^l^	1.49, 2.40	< 0.001
G6PD status unknown	47	1.07	0.94, 1.23	0.291	7	1.34	1.09, 1.65	0.005

Multivariable regression models (logistic or normal, as appropriate) with a random intercept for the study site were fitted. Random coefficient models (for primaquine dose) could not be reliably evaluated for binary outcomes and for the absolute change in Hb concentration in patients with G6PD deficiency due to small numbers. For the absolute change in Hb concentration in patients without G6PD deficiency, a random coefficient model has not improved the fit significantly and provided similar population estimates, with a population mean = 0.03 (95% CI − 0.03, 0.10) and standard deviation = 0.103 (95% CI 0.056, 0.190) for the random slope for primaquine dose (0.1mg/kg)

^a^*N* = number of observations in that category and *n* = number of patients in that category with FC < − 25% or moderate or severe anaemia, respectively

^b^Transmission intensity defined based on estimates of *P. falciparum* prevalence rate (PfPR), assuming low transmission for study sites with a PfPR < 0.15, moderate transmission if PfPR 0.15 to < 0.40, and high transmission if PfPR ≥ 0.40

^c^Includes individuals with no malaria (*n* = 224), malaria-positive or gametocytaemic at screening (*n* = 564), and symptomatic individuals from the community who were not tested (*n* = 625)

^d^For nonlinear relationship, see Additional file 3: Fig. S5

**Fig. 2:**
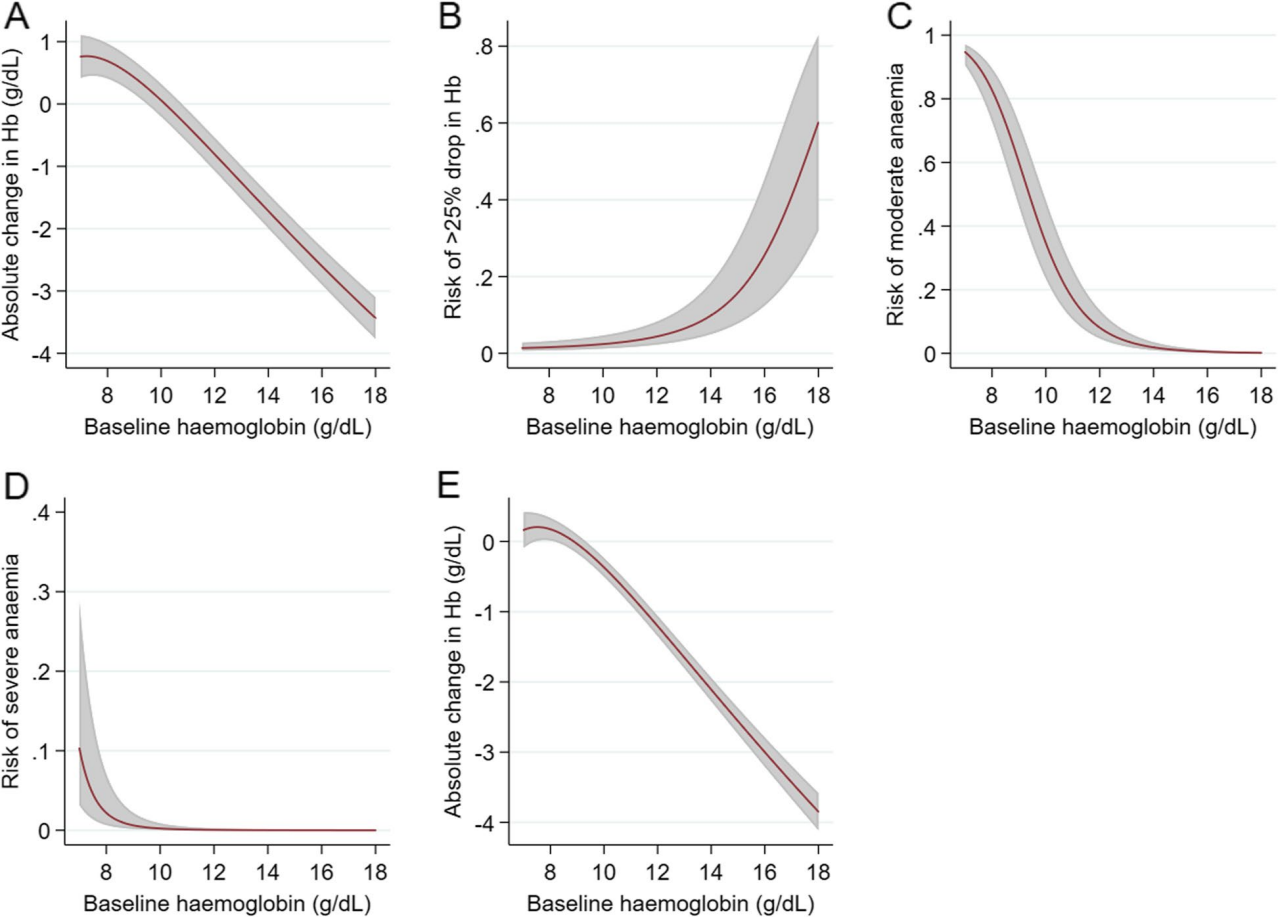
Relationship between baseline haemoglobin and haematological endpoints on day 7. The shaded area shows 95% CI. Adjusted for all independent predictors (shown in Table 3) and shown for a child < 5 years of age, from low-transmission intensity area, normal G6PD status and baseline parasitaemia = 10,000/μL. **A** Absolute change in haemoglobin on day 7 evaluated in all patients. **B** Risk of > 25% drop in haemoglobin on day 7. **C** Risk of moderate/severe anaemia (Hb < 10 g/dL) on day 7. **D** Risk of severe anaemia (Hb < 7 g/dL) on day 7. **E** Absolute change in haemoglobin on day 7 evaluated in patients from sub-Saharan Africa. **A**–**D** used data from all locations. For the interpretation of the association in A and E, please refer to Additional file 3: Table S3.

^e^For nonlinear relationship, see Fig. 2

^f^*N* and *n*, defined as before but refer to individuals who received primaquine in that category

^g^Change in Hb = − 0.528, (95% CI − 0.887, − 0.168), *P* = 0.004, for comparison of 0.25 mg/kg primaquine (83) and no primaquine arms (67) in patients with G6PD deficiency

^h^AOR = 3.98, (95% CI 0.77, 20.59), *P* = 0.099, for comparison of 0.25 mg/kg primaquine (8/83) and no primaquine arms (2/67) in patients with G6PD deficiency, only adjusted for haemoglobin due to small numbers

^i^Adjusted only for baseline haemoglobin levels due to small numbers

^k^AOR = 3.25, (95% CI 0.96, 10.95), *P* = 0.057, for comparison of 0.25 mg/kg primaquine (17/83) and no primaquine arms (11/67) in patients with G6PD deficiency

^l^AOR = 10.36, (95% CI 0.28, 389.51), *P* = 0.206 for comparison of 0.25 mg/kg primaquine (2/83) and no primaquine (1/67) arms in patients with G6PD deficiency, not adjusted for the site due to small number of events observed

Regardless of primaquine administration, young age (compared to males aged 12 and above) was associated with larger reductions in haemoglobin concentration and therefore a higher risk of anaemia (adjusted odds ratio (AOR) 3.52, 95% confidence interval (CI) 2.18–5.68 for children < 5 years of age and AOR 1.88, 95% CI 1.23–2.92 for children 5–11 years old) (Table 3). The effect of parasitaemia on haemoglobin concentration was nonlinear, with a steep increase in the risk of a haemoglobin drop observed in participants with parasitaemia > 10,000/μL (Additional file 3: Fig. S5). The relationship between baseline haemoglobin and change by day 7 varied with baseline haemoglobin concentration (Fig. 2, Additional file 3: Table S3). In participants with Hb0 ≤ 9 g/dL, higher baseline levels were associated with larger absolute and fractional changes by day 7 (*P* < 0.001 for both), while in participants with Hb0 > 9 g/dL, there is little evidence for the differential changes across the levels of baseline haemoglobin values

The results for all covariates were consistent across haematological response measures (Table 3, Additional file 3: Tables S4-S5). After adjusting for baseline haemoglobin level and other significant predictors in this sample of 194 G6PD-deficient participants, a primaquine target dose of 0.25 mg/kg was associated with an additional drop in haemoglobin concentration of 0.53 g/dL (95% CI 0.17–0.89) and 3-fold increased odds of crossing the moderate anaemia threshold (< 10 g/dL; AOR 3.25, 95% CI 0.96–10.95) compared to the no primaquine group. An additional drop of 0.27 (95% CI 0.19–0.34) g/dL haemoglobin was estimated for every 0.1 mg/kg increase in primaquine dose. Similarly, each 0.1 mg/kg increase in primaquine dose was associated with an increased risk of moderate/severe anaemia (Hb < 10 g/dL; AOR 1.60, 95% CI 1.28–1.99) or severe anaemia (Hb < 7 g/dL; AOR 1.89, 95% 1.49–2.40) (Table 3).

**Fig. 3:**
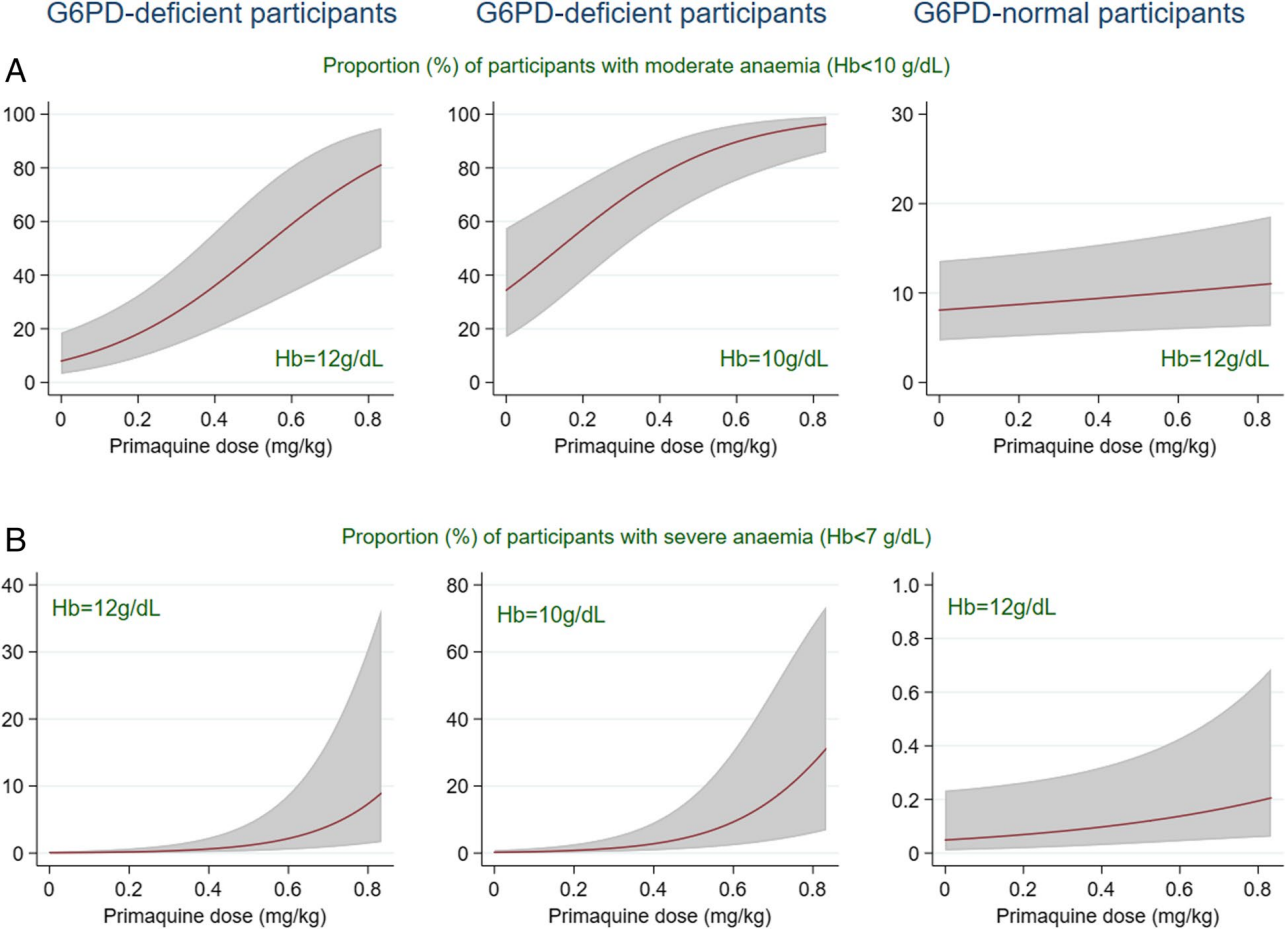
Estimated primaquine dose-response relationship. The shaded area shows 95% CI. Adjusted for all independent predictors (shown in Table 3) and shown for children < 5 years of age, from low-transmission intensity settings with parasitaemia of 10,000/μL, and for G6PD-deficient and G6PD-normal patients with different levels of baseline haemoglobin. **A** Moderate/severe anaemia (Hb < 10g/dL). **B** Severe anaemia (Hb < 7 g/dL) on day 7

The estimated dose-response curves for risk of anaemia in G6PD-deficient and normal young children (< 5 years old) from low-transmission intensity settings with a baseline parasitaemia of 10,000/μL with different baseline haemoglobin values are presented in Fig. 3 (see Additional file 3: Fig. S6 for other endpoints). No evidence for a non-linear dose-response relationship was found for the AC7 endpoint (for residual and goodness of fit plots, see Additional file 1).

#### Nadir of the haemoglobin concentration and haematological recovery

Among 1378 participants (355 with no primaquine and 1023 with primaquine) with haemoglobin measurements on days 0, 2, 3, and 7, the majority (54.9%) had a haemoglobin concentration nadir on day 2 or day 3. This was consistent regardless of whether individuals received primaquine (55.7%, 570/10,232) or not (52.7%, 187/355) and regardless of G6PD status. In the subset of 72 G6PD-deficient participants, primaquine doses were all administered on day 0.

Of the 975 participants who presented with moderate/severe anaemia (Hb < 10 g/dL) either on or after the day primaquine was administered (day 0, 2, or 3) and within 7 days of the first dose of ACT administration, 72.6% (708) had at least one subsequent measurement. These individuals were included in the analysis of recovery from anaemia (Table 4). Among these individuals, 16% (95% CI 13–18) recovered within 3 days, 54% (95% CI 51–58) within 7 days, and 76% (95% CI 73–79) within 14 days from the first day of anaemia (since primaquine administration in the study). Time to recovery was not affected by primaquine dose, timing of dose administration, or G6PD status (Additional file 3: Table S6). Recovery from anaemia took longer in young children (adjusted hazard ratio for recovery from anaemia AHR 0.72, 95% CI 0.60–0.88 compared to children 5–11 years old and AHR 0.86, 95% CI 0.65–1.14 compared to participants 12 years of age or older) and participants with hyperparasitaemia (AHR 0.70, 95% CI 0.53–0.93 compared to participants with parasitaemia < 100,000/μL) (Table 4). A higher baseline haemoglobin concentration was associated with a shorter time to anaemia recovery (AHR 1.34, 95% CI 1.26–1.42 per 1g/dL increase), and participants presenting with anaemia on day 0 took longer to recover than participants who developed anaemia after the initiation of treatment with ACT (Additional file 3: Fig. S7), even after adjusting for the haemoglobin level when anaemia was first observed (and age and baseline hyperparasitaemia) (AHR 0.56, 95% CI 0.46–0.67, *P* < 0.001).

**Table 4:** Predictors of recovery from anaemia, multivariable Cox regression model with shared frailty term for study site

	*N*	*n*	AHR	95% CI	*P*-value
Baseline Hb (per 1 g/dL increase)	698	592	1.34	1.26, 1.42	< 0.001
Age					
< 5 years	265	215	1.00		
5–11 years	285	255	1.38^a^	1.14, 1.68	0.001
12+ years	148	122	1.17^b^	0.88, 1.55	0.281
Hyperparasitaemia^c^					
Yes	75	66	0.70	0.53, 0.93	0.012
No	623	526	1.00		

*N* number evaluated, *n* number with recovery observed

^a^AHR 0.72, 95% CI 0.60–0.88 for comparison < 5 years old to 5–11 years old

^b^AHR 0.86, 95% CI 0.65–1.14 for comparison < 5 year old to 12+ years old

^c^Defined as parasitaemia > 100,000/μL and compared to patients with lower parasitaemia density (*n* = 468), community participants with no detectable parasitaemia (*n* = 23) or study participants with no detectable parasitaemia on enrolment but malaria-positive or gametocytaemic at screening (*n* = 132)

Haemoglobin data of participants who experienced anaemia at Hb < 7 g/dL at any point during the study are listed in Additional file 3: Table S7. Among the 77 participants with Hb < 7 g/dL reported at any time point (including enrolment), only 37 (48.1%) had some follow-up information available; 29 (78.4%) of them recovered from severe anaemia within 7 days, and 8 (21.6%) were still severely anaemic after 7 days, with no further follow-up data available. Among these 77 individuals, only two had Hb < 5 g/dL: the first was a G6PD-normal 4 year-old who did not receive primaquine (baseline 5.3 g/dL Hb that dropped to 4.9 g/dL 7 days later with no further follow-up data), and the second was a 7-year-old G6PD-normal individual who received 0.75 mg/kg primaquine (Hb 8.3 g/dL at baseline, which dropped to 4.7 g/dL on day 3 and recovered to 10.2 g/dL on day 14).

### Analysis of adverse events

Eleven studies with 3629 participants, 85% (3 113) of whom received primaquine, had line listings of AE IPD available: 8 controlled studies (1143 participants) and 4 uncontrolled studies (1970 participants). One study (ID 9) had both controlled and uncontrolled parts (Table 5). Baseline characteristics of participants in the AE IPD analysis were similar to those of the entire dataset, with similar proportions of G6PD-deficient and hyperparasitaemic in primaquine and no primaquine arms (Additional file 3: Table S8).

**Table 5:** Summary of adverse events by time since primaquine dosing

	Controlled studies				Uncontrolled studies	Total
	Primaquine, ***N*** [%] with AE	No primaquine, ***N*** [%] with AE	OR (95% CI)^a^	***P***-value	Primaquine^**d**^, ***N*** [%] with AE	Primaquine, ***N*** [%] with AE
**By day 28**	7 studies 896 participants	7 studies 403 participants			2 studies 139 participants	9 studies 1035 participants
Any SAE	14 [1.6]	5 [1.2]	1.41 (0.48–4.18)	0.535	0 [0]	14 [1.4]
Any AE ≥ grade 2^b^	44 [11.5]	36 [15.8]	0.78 (0.47–1.29)	0.330	9 [6.5]	53 [5.1]
**By day 7**	8 studies 1143 participants	8 studies 516 participants			4 studies 1970 participants	12 studies 3113 participants
Any AE	238 [20.8]	99 [19.2]	1.31 (0.99–1.74)	0.062	35 [1.8]	273 [8.8]
Any SAE	10 [0.9]	4 [0.8]	1.35 (0.39–4.65)	0.635	1 [0.1]	11 [0.4]
Haematological SAE	8 [0.7]	4 [0.8]	1.14 (0.31–4.12)	0.845	1 [0.1]	9 [0.3]
Any AE ≥ grade 2^b^	48 [7.6]	35 [10.3]	0.90 (0.55–1.45)	0.655	13 [0.7]	61 [2.0]
Vomiting	14 [1.2]	10 [1.9]	0.88 (0.37–2.06)	0.761	2 [0.1]	16 [0.5]
Headache	57 [5.0]	20 [3.9]	1.26 (0.73–2.16)	0.408	6 [0.3]	63 [2.0]
Pyrexia	45 [3.9]	29 [5.6]	0.96 (0.58–1.61)	0.882	1 [0.1]	46 [1.5]
Abdominal pain	29 [2.5]	11 [2.1]	1.42 (0.68–2.96)	0.347	0 [0]	29 [0.9]
Any gastrointestinal	52 [4.5]	25 [4.8]	1.19 (0.70–2.01)	0.514	4 [0.2]	56 [1.8]
**By day 3**	8 studies 1143 participants	8 studies 516 participants			4 studies 1970 participants	12 studies 3113 participants
Any AE	166 [14.5]	76 [14.7]	1.37 (0.99–1.91)	0.061	23 [1.2]	189 [6.1]
Any SAE	8 [0.7]	4 [0.8]	1.15 (0.32–4.10)	0.834	1 [0.1]	9 [0.3]
Haematological SAE	6 [0.5]	4 [0.8]	0.93 (0.24–3.58)	0.917	1 [0.1]	7 [0.2]
Any AE ≥ grade 2^b^	37 [5.9]	31 [9.1]	0.83 (0.49–1.42)	0.498	9 [0.5]	46 [1.5]
Haemoglobinuria^c^	39 [2.6]	13 [1.9]	2.39 (1.22–4.67)	0.011	1 [0.1]	53 [1.8]
Vomiting	13 [1.1]	10 [1.9]	0.83 (0.35–1.98)	0.676	2 [0.1]	15 [0.5]
Headache	35 [3.1]	15 [2.9]	1.09 (0.57–2.07)	0.792	4 [0.2]	39 [1.3]
Pyrexia	34 [3.0]	25 [4.8]	0.97 (0.55–1.72)	0.928	1 [0.1]	35 [1.1]
Abdominal pain	19 [1.7]	8 [1.6]	1.56 (0.65–3.73)	0.321	0 [0]	19 [0.6]
Any gastrointestinal	40 [3.5]	21 [4.1]	1.24 (0.69–2.23)	0.464	2 [0.1]	42 [1.3]

*N* number of participants

^a^Adjusted for study site

^b^Grading not available for one study (ID 2) with 288 AEs and a further 30 AEs from another 2 studies, all RCTs (primaquine: day 28, 145; day 7, 85; day 3, 39; no-primquine: day 28, 48; day 7, 27; day 3, 11)

^c^Eight studies had haemoglobinuria data in controlled studies, 1516 participants in the primaquine arm and 680 in the no primaquine arm. These data include 19 records of haemoglobinuria that were not reported as AEs. One study with 813 participants had haemoglobinuria data in uncontrolled studies. There was no haemoglobinuria reported after day 3

^d^Exact day of SAE within 7 days of follow-up not known for one haematological SAE in uncontrolled studies

Among 11 studies with available AE data, 273/3113 (8.77%) participants taking primaquine had at least one AE within 7 days of primaquine administration and 189/3113 (7.07%) within 3 days. The most prevalent AEs were gastrointestinal disturbance, headache, and pyrexia (Table 5). The number and proportion of AEs were lower in uncontrolled studies where all participants received primaquine. In controlled studies, with the exception of haemoglobinuria (described below), the proportions of any AE and SAE were similar between the primaquine and no primaquine arms, as was any event of grade 2 and above.

Additional file 3: Table S9 contains details of all SAEs (27 in 19 patients) emerging after the primaquine dosing day until day 28. In the eight controlled studies, 0.8% of the participants in both the primaquine (9/1143) and non-primaquine (4/516) arms developed haematological SAEs and twelve occurred within 7 days of primaquine administration. Six (three in the 0.25 mg/kg primaquine arm including two G6PD-deficient patients and three in the no primaquine arm) came from one study meeting a predefined SAE criterion of acute haemolytic anaemia. None required medical intervention, and all recovered within 2 days. A further six haematological SAEs in another study were due to a trial-specific predefined SAE criterion relating to drops in haemoglobin concentration of > 3g/dL: five within 7 days of dosing primaquine ≥ 0.25 mg/kg, all considered possibly related to the study drug, and one in the no primaquine arm. Haemoglobin levels for five participants (four G6PD-normal and one G6PD-deficient) were consistently rising from the next visit (3 or 7 days later). The sixth participant (0.25 mg/kg primaquine, G6PD-normal) had a sustained drop of 3 g/dL haemoglobin from 11.8 g/dL at baseline within a day of primaquine dosing, considered possibly related to the study drug. She was an underweight 2-year-old who also presented with haemoglobinuria, cough, diarrhoea, and pyrexia and who was given a blood transfusion on day 14, after which her condition resolved. The thirteenth haematological SAE was haemoglobinuria in a female aged 35 years given 0.25 mg/kg primaquine on day 1. She was G6PD-normal by FST but heterozygous for the Mahidol mutation, a beta-thalassaemia carrier, and was subsequently diagnosed with human immunodeficiency virus (HIV) and tuberculosis. Her date of onset of haemoglobinuria, its relation to the study drug, and outcome were unclear.

Two deaths reported in an observational study were deemed unrelated to primaquine by the primary study authors. Symptoms in one who was diagnosed with uncomplicated *P. falciparum* malaria, treated with artemether-lumefantrine and 0.25 mg/kg of primaquine and died 2 days later were attributed to a pre-existing unspecified immune-compromised condition. A second individual with malaria symptoms was treated based on signs and symptoms of malaria with 0.25 mg/kg primaquine and artemether-lumefantrine despite being malaria-negative by RDT and microscopy. His condition deteriorated, and he died 10 days later from an unknown cause.

#### Haemoglobinuria

Eight controlled studies (2184 participants) were included in the hemoglobinuria analysis. G6PD deficiency was detected in 141 (6.5%) participants (all in Africa), and 133 (6.2%) had hyperparasitaemia (Additional file 3: Table S10). Primaquine was administered to 69% (1507) of participants (dose range 0.0625–0.75 mg/kg) with 27% (410) and 73% (1106) initiated on primaquine at day 0 and day 2, respectively. All G6PD-deficient participants received primaquine on day 0.

A total of 52 (2.4%) episodes of haemoglobinuria were reported from five studies, all within 72 h of primaquine treatment (Table 5). Haemoglobinuria was more frequent in the primaquine arm within 3 days of primaquine dosing (OR 2.39, 95% CI 1.22–4.7), but no haemoglobinuria was observed after day 3 in either arm of controlled studies (Additional file 3: Table S11, Fig. S8). Except for three participants who reported haemoglobinuria on two consecutive days in one study (study ID 11), there were no reports of haemoglobinuria persisting longer than 1 day. A higher incidence of haemoglobinuria was observed in participants with G6PD deficiency (adjusting for primaquine administration) compared to participants with normal G6PD status (6/47, 11.3% compared to 7/615, 1.1% in no primaquine arms and 14/88, 15.9% compared to 25/1416, 1.8% in primaquine arms). Participants who received a 0.25 mg/kg target dose of primaquine had a higher risk of haemoglobinuria (OR = 2.44, 95% CI 1.25–4.77) than those who did not receive primaquine The risk of haemoglobinuria increased with primaquine dose (AOR 1.36, 95% CI 1.08–1.72 per 0.1 mg/kg increase) and, after adjusting for dose, was higher in participants with hyperparasitaemia (AOR 3.91, 95% CI 1.38–11.12) or G6PD deficiency (AOR 2.30, 95% CI 1.15–4.60) (Table 6). We found no significant interaction between G6PD status and either primaquine administration or primaquine dose. There were also no associations between age, sex, baseline haemoglobin concentration, and risk of haemoglobinuria in both univariable (Additional file 3: Table S11) and multivariable analyses (Table 6).

**Table 6:** Risk factors for haemoglobinuria after treatment, multivariable logistic regression model with a random intercept for study site

	*N*	*n*	AOR	95% CI	*P*-value
G6PD status					
Normal	2038	32	1.00		
Deficient	141	20	2.30	1.15, 4.60	0.018
Dose of PQ^a^ (per 0.1 mg/kg)	2179	52	1.36	1.08, 1.72	0.010
Hyperparasitaemia^b^					
No	2046	45	1.00		
Yes	133	7	3.92	1.38, 11.12	0.010

*N* number evaluated, *n* number with haemoglobinuria

^a^AOR = 2.47, 95% CI 1.25–4.89, *P*-value = 0.009, for comparison between target 0.25 mg/kg primaquine dose and no primaquine arms

^b^Defined as parasitaemia > 100,000/μL; compared to patients with lower parasitaemia (*n* = 1468), no detectable (*n* = 523) or unknown parasitaemia (*n* = 55) in study participants who were malaria-positive or gametocytaemic at screening

#### Other adverse events of special interest

Non-haematological SAEs in controlled studies were all considered unrelated or unlikely to be related to primaquine by the primary study authors according to pre-defined causality assessment methods, except for vomiting in a 4-year-old on dosing day (primaquine 0.125 mg/kg) that was considered possibly related to primaquine (Additional file 3: Table S9). There were no statistically significant differences between primaquine and no primaquine arms for any gastrointestinal AE, abdominal pain, or vomiting by day 3 or day 7 (Table 5).

#### Simulation of risk of haemoglobin concentrations below 7 and 5 g/dL on day 7 in sub-Saharan African participants with G6PD deficiency

Coefficient estimates in the multivariable model for absolute change in haemoglobin concentration on day 7 (AC7) in sub-Saharan Africa (Additional file 3: Table S12, Fig. S5, Fig. 2) were very similar to the coefficients in the main model presented in Table 3. Variability between studies and between participants decreased when the analysis was restricted to African participants (from *σ*_*u*_ = 0.50 and *σ*_*e*_ = 1.16 to *σ*_*u*_ = 0.25, and *σ*_*e*_ = 1.13, respectively). The distribution of baseline parasitaemia used in the simulation is shown in Additional file 3: Fig. S9.

**Fig. 4:**
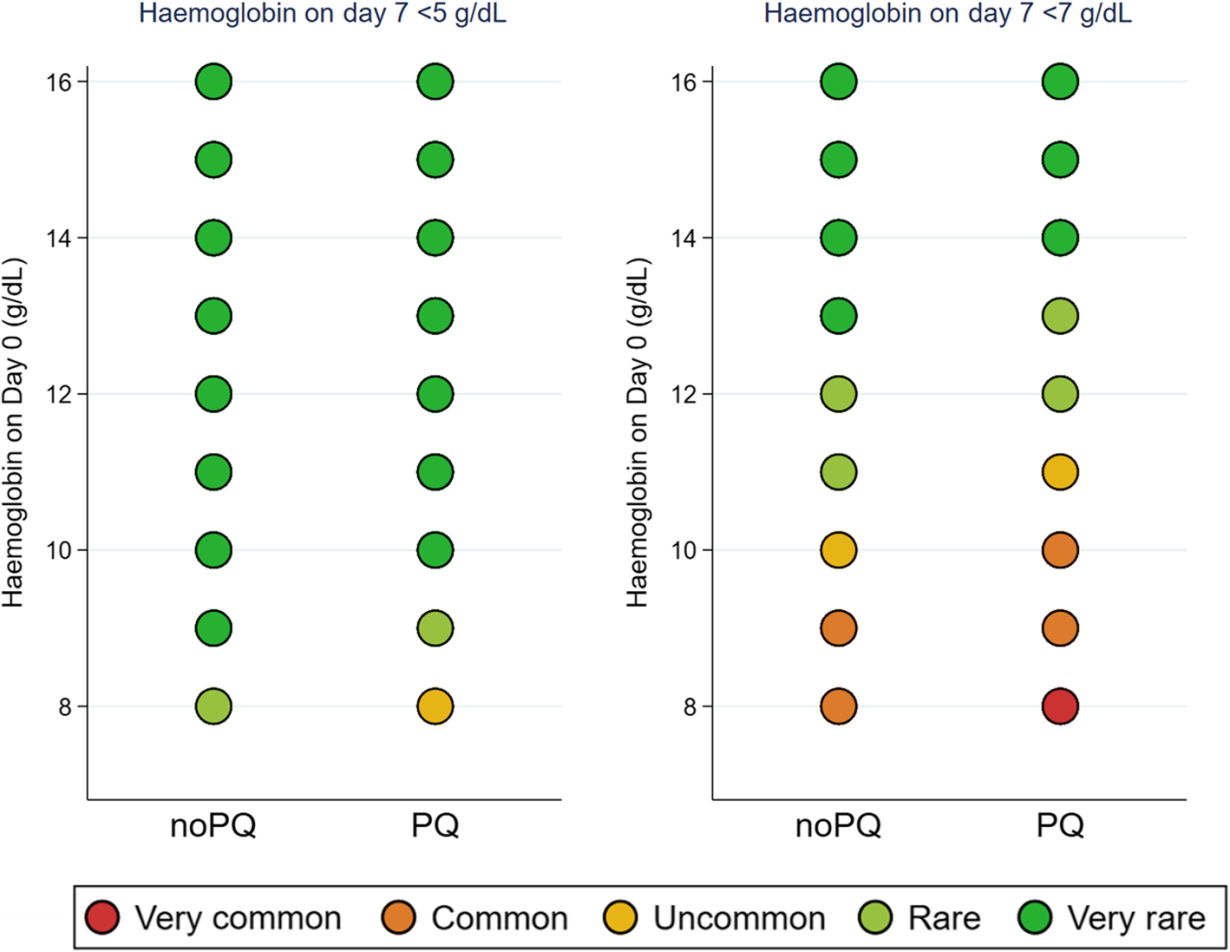
Classification of predicted frequency of severe anaemia (Hb < 7 g/dL, Hb < 5 g/dL) on day 7. Shown for *Plasmodium falciparum* malaria patients with G6PD deficiency in sub-Saharan Africa, treated with ACT (noPQ) or ACT + 0.25 mg/kg primaquine dose (PQ) shown for each value of baseline haemoglobin concentrations observed in G6PD-deficient population (*y*-axis). The results come from 100,000 simulated day 7 haemoglobin concentrations for each day 0 haemoglobin concentration value, age group, and transmission intensity from the AC7 model presented in Additional file 3: Table S12. Classification of AE frequency: very common ≥ 1/10, common ≥ 1/100 and < 1/10, uncommon ≥ 1/1000 and < 1/100, rare ≥ 1/10,000 and < 1/1000, and very rare < 1/10,000

As expected, the simulated risk of anaemia among individuals with G6PD deficiency varied substantially with baseline haemoglobin concentration (Fig. 4, Additional file 3: Figs. S10-S11). A G6PD-deficient individual with baseline haemoglobin level *x* g/dL who received 0.25 mg/kg of primaquine had a similar risk of severe anaemia (Hb < 7 g/dL) to an individual who had not received primaquine with a 1 g/dL lower (*x* − 1 g/dL) baseline haemoglobin level. In G6PD-deficient individuals given 0.25 mg/kg primaquine, severe anaemia was uncommon (between 1/100 and 1/1000) for individuals with baseline haemoglobin concentration ≥ 11 g/dL but common (1/10 to 1/100) for baseline haemoglobin concentrations between 9 and 10 g/dL. The risk of anaemia in this group that is considered likely to lead to blood transfusion (Hb < 5 g/dL) was very rare (≤ 1/10,000) if baseline haemoglobin concentration was ≥ 10 g/dL, rare (between 1/1000 and 1/10,000) for baseline haemoglobin of 9 g/dL, and uncommon for baseline haemoglobin 8 g/dL, compared to very rare for haemoglobin ≥ 9 g/dL and rare for haemoglobin of 8 g/dL in the no primaquine group.

Additional file 3: Figs. S10-S11 show the estimated risk of anaemia for different age groups and primaquine doses of 0, 0.25, and 0.4 mg/kg. Risk of anaemia (Hb < 7g/dL or Hb < 5g/dL) for a participant with a baseline haemoglobin level *x* g/dL who received a 0.4 mg/kg dose is slightly higher than for a participant with the same baseline haemoglobin level *x* g/dL who received a 0.25 mg/kg dose, and much lower than for a participant with (*x* − 1) g/dL haemoglobin level, who received a 0.25 mg/kg dose.

### Risk of bias

See Additional file 4: Table S13 for the factors considered when assessing the risk of bias. All RCTs used random sequence number generation methods and sealed, opaque envelopes, so were at a low risk of selection bias. Most studies were open-labelled, except for three where study personnel and participants were blinded to allocation, one where only participants were blinded, and one where participants could be unblinded on request by a non-trial staff member. In four studies, all the staff appear to be blinded, and in two, the laboratory staff were blinded but the clinical staff were not. The lack of blinding is considered unlikely to affect the primary haematological outcome but increases the risk of bias in reporting AE results. Similarly, variability in methods for determining AEs between studies increases the risk of reporting bias; however, comparison between arms should be less affected as was done within studies, and study arms followed the same protocol. Data for the primary day 7 haemoglobin outcome were available for over 90% of participants in 50% of the studies (9/18), between 80 and 89% in a further three studies, and between 60 and 79% in the remaining six studies. Sensitivity analysis for the effect of primaquine dose on the main haematological outcomes (absolute change in haemoglobin concentration on day 7 and risk of moderate/severe anaemia on day 7) did not identify significant evidence of bias related to the included studies (Additional file 4: Fig. S12). Safety data and haematological response in the 4 studies for which IPD data were not available for this analysis were very similar to the data presented in this report (for details, see Additional file 4: Table S14).

## Discussion

Malaria causes the loss of erythrocytes and thus poses an increased risk of crossing the thresholds for the definition of anaemia. Iatrogenic haemolysis may further compound this risk. This IPD meta-analysis of 6406 participants in Africa and Asia estimated the haemolytic effects of SD primaquine (target dose 0.0625 to 0.75 mg/kg) when administered with an ACT. Our study findings show that a lower baseline haemoglobin concentration is the strongest predictor of the risk of anaemia (i.e., the closer the individual is to the anaemia threshold value before treatment, the greater the likelihood of crossing that threshold). In G6PD-normal individuals, this risk of anaemia 7 days after receiving the first dose of ACT was not associated with SD primaquine use. When a 0.25 mg/kg dose of SD primaquine was given to a G6PD-deficient individual, the fall in haemoglobin increased by an average of 0.53 g/dL (95% CI 0.17–0.89). Higher doses of primaquine resulted in additional haemoglobin drops of approximately 0.27 (05% CI 0.19–0.34) g/dL per 0.1 mg/kg increase in primaquine dose.

Although our study found an increased risk of anaemia in 194 G6PD-deficient individuals who received SD primaquine, haemoglobin dropped below 7g/dL (Hb0 − Hb7: 8.2–6.8, 8.8–6.0 g/dL) in only two patients, and serious adverse haematological events were few and transitory. Among 13 haematological SAEs (nine G6PD-normal and four G6PD-deficient) in controlled studies by day 7 (9 of whom received primaquine), 11 recovered without further intervention. One received a blood transfusion after which her condition resolved; she was an underweight G6PD-normal 2-year-old who received 0.25 mg/kg primaquine. The other was a G6PD-deficient woman (heterozygous for the Mahidol mutation) diagnosed with HIV and tuberculosis who had received 0.25 mg/kg of primaquine and developed haemoglobinuria; its relationship to primaquine and AE outcome were unclear.

Our study confirms previously identified risk factors for the onset of anaemia. In addition to baseline haemoglobin being the most important predictor of anaemia, parasite densities > 10,000/μL were associated with increased reductions in haemoglobin concentration, as malaria infection can cause haemolysis. Unfortunately, in the normal context of use, parasite density is seldom known, as a definitive malaria diagnosis is usually made only with a rapid diagnostic test. Young age was associated with a higher risk of anaemia, with those < 5 years of age having over threefold the risk of anaemia compared to males 12 years and older after adjusting for baseline haemoglobin and parasite load. Frequency of very severe anaemia (Hb < 5 g/dL) among G6PD-deficient participants receiving 0.25 mg/kg of primaquine was very rare (≤ 1/10,000) if baseline haemoglobin was ≥ 10 g/dL, rare (between 1/1000 and 1/10,000) if baseline haemoglobin was 9 g/dL, and uncommon (between 1/100 and 1/1000) if baseline haemoglobin was 8 g/dL. These findings suggest that a target primaquine dose of 0.25 mg/kg is safe for implementation, including in G6PD-deficient individuals, provided they are not severely anaemic at baseline. Although the risk of developing anaemia requiring blood transfusion is very low, access at a reasonably nearby referral facility is recommended for the very rare occasions when needed [12].

Most participants in this analysis had a nadir in haemoglobin concentration on days 2 or 3 following the first ACT dose. Anaemia (Hb < 10 g/dL) was found to be transient, with 54% of cases recovering in 7 days and 76% within 14 days. A similar result was found among cases of severe anaemia (Hb < 7 g/dL) with 78% recovering within 7 days, but limited follow-up data were available.

Reassuringly, the proportion of participants in controlled studies who experienced any AE or SAEs were similar between those who received primaquine and those who did not, as were any event of grade 2 or above. Other than haematological safety discussed above, AEs comprised primarily malaria-related symptoms (such as fever and headache) and gastrointestinal AEs, of which vomiting is considered most clinically significant as this may impair antimalarial drug absorption and thus therapeutic efficacy. The risk of these AEs was similar between the primaquine and no primaquine arms in controlled studies, with vomiting reported in 1.2% of those randomised to primaquine and 1.9% of those randomised to no primaquine. Gastrointestinal AEs can be reduced by administering primaquine with food [3].

Our findings are confined to African and Asian settings, with African settings representing 91.7% of the IPD on haematological response and all day 7 data in G6PD-deficient participants. This has important implications on the generalisability of this study. Although we did not identify the variants of G6PD deficiency, these can be characterised geographically, with estimated frequencies of 8% across malaria-endemic countries (from < 1 to 23%) [13, 14]. In sub-Saharan Africa, variants of G6PD-deficiency are generally less severe and are similar to those found in South and Central American settings, where we would expect similar effects of primaquine to those found in our study. For Asian settings, our analysis only included Indonesian haemoglobin data for G6PD-normal participants and AE data from two studies in Thailand and Bangladesh. Our findings are thus not as readily transferable to west Asian settings, where the severe Mediterranean variant of G6PD deficiency is dominant. In these settings, evidence from old MDA studies to eradicate vivax malaria conducted in over 7 million people suggests that 14-day 210 mg total dose of primaquine is safe [15]. However, caution should be undertaken in these locations, as the only historically documented death from acute haemolysis following the use of a single higher dose of primaquine (45 mg; 0.75 mg/kg) was in a man in India with the Mediterranean variant of G6PD deficiency [3].

After the cut-off date for inclusion in this analysis, several additional studies on SLD primaquine have been published which support our findings, namely that 0.25 mg/kg of primaquine is well tolerated in sub-Saharan African and Southeast Asian settings, with no associated severe haemolysis, or SAEs related to study drugs. Two of these studies used 0.25 mg/kg of primaquine with DP as part of mass drug administration activities reaching over 10,000 individuals in Zanzibar [16], and 8445 participants in Myanmar, Vietnam, Cambodia, and the Lao People’s Democratic Republic, although Cambodian participants were not given primaquine for regulatory reasons [17]. Two Cambodian studies have since been published; however, one efficacy study (109 participants) [18] and one open randomised trial on primaquine tolerability (109 participants, 12 were G6PD-deficient with the Viangchan variant) show a 0.25 mg/kg dose of primaquine to be reasonably safe (in 11 G6PD-deficient patients, day 7 haemoglobin concentration dropped from 8.2 to 7.5g/dL in one participant, for two remained below 8g/dL with small changes [6.9–6.4, 7.4–7.4 g/dL], and stayed above 8g/dL for the remaining nine). This informed the Cambodian national decision to deploy 0.25 mg/kg of primaquine with ACTs nationwide in 2018 [19].

National malaria control programmes considering the use of SLD primaquine can apply our findings which, when combined with results from the studies cited above, suggest that a single primaquine target dose of 0.25 mg/kg is generally safe and well-tolerated when given together with an ACT. Higher doses of 0.40 mg/kg primaquine were also well tolerated, although leading to slightly higher risks of haemolytic drops among G6PD-deficient individuals. The additional drops in haemoglobin resulting from higher doses of primaquine are transient and considered relatively small, as baseline haemoglobin concentrations are the strongest driving factor of the risk of developing moderate or severe anaemia.

This retrospective analysis was constrained by several limitations of the data. The actual primaquine dose in mg/kg was only available for some studies; for others, target dose was assumed from the manuscript/protocol; and in one study, target dose had to be estimated, as no weight data were available. There were also a relatively small number of participants in the G6PD-deficient group, with limited information on G6PD variants provided and most of these data were in African participants. The data was particularly sparse in G6PD-deficient children less than 12 years old, with only 30 G6PD-deficient children treated with primaquine (12 with 0.25mg/kg, 18 with 0.75 mg/kg dose). The original studies contributing to this IPD meta-analysis excluded patients below a lower limit of haemoglobin concentration (mostly 8 g/dL, and two studies at 11 g/dL). The limited number of participants with G6PD deficiency particularly in Western Asia, narrow baseline haemoglobin range and inexact dose information particularly impacted the quantification of the primaquine dose-response relationship in individuals who are at the highest risk of large haematological changes. There was also limited follow-up data for severe anaemia episodes; only 48% of those with haemoglobin below 7 g/dL had follow-up measurements. Adverse events monitoring varied between studies; therefore, any comparisons between primaquine and no primaquine groups were conducted only in RCTs.

As our findings represent data from clinical studies, vulnerable populations are under-represented, in particular, individuals with severe anaemia at baseline (i.e. age, sex, pregnancy, and recurrent malaria-related) and those with co-morbidities (e.g. malnutrition, helminth infections), who are usually excluded from studies on uncomplicated malaria treatment. For those known to be at increased risk of haemolysis, which we identified as having G6PD deficiency, severe anaemia at baseline, high parasite density (> 10,000/μL), and young age (< 5 years), risk mitigation could include patient (caregiver) information on monitoring for signs and symptoms of haemolysis for a week following primaquine administration. Evidence from this analysis is insufficient to capture rare events (< 1/1000) with the use of single-dose primaquine to interrupt transmission of *P. falciparum* malaria. Therefore, as with any new treatment policy deployment, post-marketing pharmacovigilance should be encouraged, for which existing tools are available [9, 12, 20]. This could help address these real-world evidence gaps, as well as identify any effects of inadvertent exposure to SLD primaquine during pregnancy (when primaquine is contraindicated).

## Conclusions

Both the IPD meta-analysis on efficacy and this safety IPD meta-analysis, support the WHO recommendation on SLD primaquine in elimination settings and areas threatened by artemisinin resistance to reduce *P. falciparum* transmission from treated infections. G6PD testing is not recommended given that primaquine is associated with a mean 0.53g/dL drop in haemoglobin in G6PD-deficient individuals. Although primaquine is associated with a transient reduction in haemoglobin levels in G6PD-deficient individuals, haemoglobin levels at baseline remain the major drivers for anaemia in these patients.

## Methods

The primary objectives of this study were to quantify absolute and fractional reductions in haemoglobin concentration (Hb) associated with the addition of a range of SD primaquine doses to an ACT blood schizonticide between days 0 and 7 after ACT administration (and their potential predictors), the proportion of participants with moderate/severe (defined here as Hb < 10g/dL) or severe anaemia (defined here as Hb < 7g/dL) on day 7 and a > 25% drop in haemoglobin between baseline and day 7, as defined previously [11, 21]. We were also interested in determining the proportion of participants whose haemoglobin level dropped below 5g/dL (defined here as very severe anaemia), a threshold often used in Africa for blood transfusion [22]. Secondly, we aimed to determine the incidence of adverse events (AEs), including serious AEs (SAEs), blood transfusions, AEs of grade 2 and above (within 7 and 28 days of primaquine administration), and other AEs of special interest (haemoglobinuria, as a marker for haemolysis, and gastrointestinal AEs within 7 days), and their association with primaquine administration. Trials or prospective cohort of adults and/or children (including mass drug administration, MDA, studies), in patients with uncomplicated *P. falciparum* infection or aparasitaemic, who received an ACT and primaquine, at a target dose of 0.75 mg/kg or less and collected haemoglobin or haematocrit levels (days 0 and 7) and/or safety data were eligible for inclusion. Besides safety data, age, treatment information and primaquine target mg/kg dose were the minimum data required for inclusion. Throughout this analysis, day 0 is defined as the day of the first ACT dose [23].

### Study identification and selection, data collection, and management

The search of studies was conducted through MEDLINE, Web of Science, and Embase platforms using the terms “(Single OR low) AND dose AND primaquine”, with no language restrictions, for studies published between 01 January 2007 and 31 May 2018. Trial registries and relevant national programmes were also explored. Additional details are provided in Additional file 1. The 2007 lower cut-off date was chosen as access to IPD and documentation such as data dictionaries and protocols was expected to be more difficult for older studies. The authors of eligible studies were invited to share IPD and information on study design and methods with the WorldWide Antimalarial Resistance Network (WWARN) repository through a secure portal. IPD were standardised using the WWARN Data Management and Statistical Analysis Plan for clinical data [24]. Data queries were resolved with contributors during the curation process.

### Statistical analyses

A statistical analysis plan (SAP) was developed a priori, and Stata 15.1 was used for the analyses. Changes in haemoglobin concentration between days 0 and 7 were quantified by absolute change (AC7 = Hb7 − Hb0) and fractional change (FC7 = (Hb7 − Hb0)/Hb0 × 100%), where Hb*i* denotes haemoglobin concentration measured on day *i*. Bivariable (adjusted for baseline haemoglobin concentration value) and multivariable regression analyses of AC7 (normal), risk of FC7 < − 25% (logistic) and risk of moderate or severe anaemia on day 7 (logistic) were performed with random study-site intercept to account for clustered data. The risk of Hb < 5 g/dL was not modelled due to a very small number of events observed. Haematological recovery during follow-up was examined in a subgroup of participants with clinically relevant anaemia (Hb < 10 g/dL) at enrolment or within the first week of treatment and measured by time to the first subsequent visit when Hb > 10g/dL (Cox regression with shared frailty for study-site).

The following pre-defined covariates were evaluated: mg/kg dose of primaquine, age, sex, weight, G6PD phenotype status (normal, deficient or unknown as per primary study classification), ACT, malaria status by microscopy, baseline *P. falciparum* parasitaemia, fever, haemoglobin (haematocrit) concentration and nutritional status on enrolment, transmission intensity, region, and study design [25–27] (definitions in Additional file 1). Haemoglobin concentration levels on day 7 were then simulated from the final multivariable AC7 model and the proportion of participants expected to experience haemoglobin drop below 7 g/dL, a level of concern for programme managers, and below 5 g/dL, a common threshold for blood transfusion in malaria-endemic regions of Africa, were estimated for a range of starting haemoglobin values and primaquine doses. Details on model building and the simulation study are provided in Additional file 1. The regression between the change from baseline and the baseline value suffers from the mathematical coupling and regression to the mean problems [28]; therefore, the relationship between the change in haemoglobin on day 7 and haemoglobin on day 0 was further explored. The difference (Hb7 − Hb0) was correlated against the mean of baseline and day 7 haemoglobin values (Hb7 + Hb0)/2 (Oldham method, [29]), and the correlation between baseline value and the absolute and fractional change were tested against the appropriate (adjusted) null value [28, 29]; for details, see footnotes of Additional file 3: Table S3. Only G6PD-normal participants contributed to this part of the analysis.

Analyses of treatment-emergent AEs within 3, 7, and 28 days of primaquine administration in studies with available data were based on descriptive summaries of frequency, seriousness, and, for SAEs, relatedness to primaquine as per primary study classification. Comparisons of AE incidence between the primaquine and no primaquine arms were performed only for data from randomised controlled trials using logistic regression with a random intercept for the study site.

The risk of bias in included studies was assessed by considering randomisation sequence generation, treatment allocation concealment, blinding of outcomes assessors and participants, completeness of primary outcome data, completeness of follow-up, differences in methods, and generalisability.

### Data integrity, study group governance, and ethics

The study group comprised a coordinating team and principal investigators (and/or their designees) who contributed relevant datasets of which they retained ownership. The study group collectively made decisions about eligibility, data analysis, and publication (WWARN Publication Policy updated 2019). Data were obtained in accordance with laws and ethical approvals applicable to the countries in which studies were conducted and were fully anonymised before or during upload to the WWARN repository. The Oxford University Research Ethics Committee does not require a review of the use of existing data that are fully anonymised and that cannot be traced back to individuals.

## Supplementary Information

**Additional file 1:.** Details of Methodology.

**Additional file 2: List L1.** List of included studies. **Table S1.** Summary of characteristics of included studies.

**Additional file 3: Table S2.** Baseline characteristics of patients in the analysis of haematological response. **Table S3.** Association between haemoglobin change and baseline value. **Table S4.** Bi-variable analysis of factors associated with drop in haemoglobin on Day 7. **Table S5.** Bi-variable analysis of factors associated with anaemia on Day 7. **Table S6.** Univariable analysis of recovery from anaemia. **Table S7.** Haemoglobin measurements in patients with severe anaemia at any time. **Table S8.** Baseline characteristics of patients included in the analysis of adverse events. **Table S9.** Listing of Serious Adverse Events reported in any study. **Table S10.** Baseline characteristics of patients included in the analysis of haemoglobinuria. **Table S11.** Univariable analysis for haemoglobinuria. **Table S12.** Multivariable model of change in haemoglobin on Day 7 in sub-Saharan Africa. **Fig. S1.** Map of study sites. **Fig. S2.** Haemoglobin in G6PD deficient patients not included in the Day 7 analysis. **Fig. S3.** Relationship between haemoglobin at baseline and Day 7 as absolute change. **Fig. S4.** Relationship between haemoglobin at baseline and Day 7 as fractional change **Fig. S5.** Relationship between baseline parasitaemia and haematological endpoints. **Fig. S6.** Dose response relationship for haematological endpoints. **Fig. S7.** Time to recovery by day when anaemia was first recorded. **Fig. S8.** Meta-analysis of odds of haemoglobinuria after PQ administration. **Fig. S9.** Distribution of parasitaemia (observed) used in the simulation study. Figure S10- Predicted risk of severe anaemia (Hb<7g/dL) on Day 7. **Fig. S11.** Predicted risk of very severe anaemia (Hb<5g/dL) on Day 7.

**Additional file 4: Table S13.** Risk of bias assessment. **Table S14.** Individual participant data not available. **Fig. S12.** Sensitivity analysis for the main haematological results.

## Data Availability

The dataset(s) supporting the conclusions of this article and materials such as the protocol, tools, and ethics approval letters are available in the WWARN repository [www.wwarn.org] which can be requested through the Data Access Committee coordinated by WHO-TDR.

## Abbreviations

AC: Absolute change
ACT: Artemisinin-based combination therapy
AEs: Adverse events
AHR: Adjusted hazard ratio
AL: Artemether-lumefantrine
AP: Aartemisinin-piperaquine
AOR: Adjusted odds ratio
ASAQ: Artesunate-amodiaquine
ASSP: Artesunate-sulfadoxine-pyrimethamine
CI: Confidence interval
DP: Dihydroartemisinin-piperaquine
FC: Fractional change
G6PD: Glucose-6-phosphate dehydrogenase
Hb: Haemoglobin
HIV: Human immunodeficiency virus
IPD: Individual patient data
IQR: Interquartile range
MDA: Mass drug administration
RCTs: Randomised controlled trials
RDT: Rapid diagnostic test
SAEs: Serious adverse events
SAP: Statistical analysis plan
SD: Single dose
SLD: Single low-dose
WHO: World Health Organization
WWARN: WorldWide Antimalarial Resistance Network
