# Supplementary material for: Safety of single-dose primaquine as a Plasmodium falciparum gametocytocide: a systematic review and meta-analysis of individual patient data
Source: BMC Med. 2022 Sep 16;20:350. doi: 10.1186/s12916-022-02504-z (PMC9479278; doi:10.1186/s12916-022-02504-z)

Details of Methodology

1. General, data curation and statistical methods: additional details

Search strategy

Registries included: Clinicaltrials.gov, the European Union Clinical Trials register, the WHO International Clinical Trials Registry Platform, Pan African Clinical Trials Registry, and national clinical trials registries of India, Australia and New Zealand, China, Germany. Country guidelines were reviewed and revealed the following to target: Bangladesh, Ethiopia, Madagascar, Senegal, Sudan, Swaziland, Tanzania (Zanzibar), Thailand, Zimbabwe. Ethiopia was excluded as related to Plasmodium vivax only. In Senegal, Madagascar, Thailand, Zimbabwe, Tanzania (Zanzibar) activities to collect single low dose primaquine pharmacovigilance data were not currently underway (or data would not be ready in time for use).

Adverse event (AE) data curation

AEs with an onset between follow-up visits were assumed reported at the next visit and AEs/SAEs defined according to ICH E2A (ICH 1994). Raw AE terms were coded using the Medical Dictionary for Regulatory Activities (MedDRA) version 20.1 [https://www.meddra.org/), with events that could not be coded to one term split, when it was assumed severity, causality, and the onset date applied to all. Causality was standardised using the WWARN Data Dictionary as not related, unlikely, possible, probable, definitely, un-assessable/unclassified where feasible. Moderate and severe AEs were coded as grade 2 and above. Symptom charts with no accompanying AE details were not possible to be mapped. Haemoglobinuria was considered if dark urine was reported through direct questioning, or if urine dipstick showed blood or the Hillman score was ≥ 5.

Calculation of mg/kg dose

The actual mg per kg dose of PQ was calculated using number of tablets administered size of the tablet and patient weight. When any of these parameters were not available, the target mg/kg dose was assumed. For one study (Study ID 1) PQ dosing was age-based and only total dose was available (1.34 mg for < 1 years of age, 2 for 1-4 years, 4mg for 5-9, 6 for 10-14 and 8 mg for 15 years of age and older). We were able to estimate target dose for the last two age categories since they overlap with categories proposed in Taylor et al 2018 and for these participants we estimated the target dose as the median dose (For 10-14 years old (n=171) - target dose = 6/7.5 *0.26 = 0.208 for 1014 years old; 8/15*0.27 = 0.144 for 15+ years old). Participants from the other age categories were excluded from the analysis.

Transmission Intensity

The study sites were classified into 3 categories of Plasmodium falciparum malaria transmission intensity: low, medium and high malaria based on the Plasmodium falciparum parasite prevalence estimates PfPR obtained from the Malaria Atlas Project for specific location and year of study (for methodology see Gething et al. 2011). Study sites with a PfPR <0.15 were classified as low, with PfPR from 0.15 to <0.40 as moderate and with PfPR ≥0.40 as high transmission areas.

Anthropometric Indicators

Nutritional status was assessed using standardised age, weight, and sex specific growth reference according to the WHO 2006 recommendations using igrowup Stata package (WHO 2006) and was measured by weight-for-age metric. The nutritional status of a child will be given as a Z-score (WAZ) and classified as underweight as defined in the WHO guidelines (WAZ<-2).

Regression models

The final multivariable model was derived via the following steps: i) use backward stepwise variable selection procedure with p=0.05 to define *Model 1* with all continuous covariates untransformed and no

interactions; ii) test all interactions between PQ dose and categorical variables using likelihood ratio test and add any significant interactions into *Model 2*; (iii) for each continuous variable in the *Model 2* test fractional polynomials in turn (2 degrees of freedom) and include significant nonlinear terms in *Model 3*; (iv) in the stepwise forward fashion check if any covariates not in *Model 3* should be included in the final model on the basis of significant likelihood ratio test and effect on coefficients for other variables, if yes – build *Model 4* using forward stepwise variable selection procedure.

Haematological recovery

Haematological recovery was examined in a subgroup of patients with clinically relevant anaemia (Hb<10 g/dl) at enrolment or within the first week of treatment, and measured by time to recovery (Cox regression). Time to recovery was measured from the first time anaemia was recorded on days 0-3 if it occurred at the time of or after day of PQ administration in the study, or from the day of PQ administration if anaemia developed before PQ administration and was still confirmed at the time of PQ administration. Patients who recovered from anaemia by the day of PQ administration or for whom there is no information on haematological status on the day of PQ administration were excluded from the analysis. Time of the recovery was measured until the first visit when the Hb>10g/dL. If two consecutive measurements were missing before the recovery was observed, time was censored at the last measurement before the gap. If one visit was missed and recovery was recorded successively after the missed visit, mid-time was assumed for recovery. Anaemia was assumed to be present on a missed visit if it was observed on both the preceding and the following visits. Same definitions (using day of PQ administration) were applied to patients in PQ and no PQ arms, so that the two groups were comparable.

Simulation study

A small number of patients experiencing severe anaemia on Day 7 provided a limited power for the analysis of risk factors associated with the risk of severe anaemia. Therefore, a model of the absolute change in Hb on Day 7 was used to simulate changes in haemoglobin and estimate prevalence of severe anaemia in the study population on Day 7. To model more homogenous population, for the purpose of simulation study, model presented in Table 2 was refitted in patients from sub-Saharan Africa only.

Haemoglobin on Day 7 for participant i observed at study-site s was estimated using the following equation:

$$Hb7_{is} = Hb0_i + AC7_i + u_s + e_i \qquad (1)$$

where $AC7_i$ is a linear predictor from the final AC7 model (Supplementary Table 5) calculated using coefficient point estimates, $u_s \sim N(0, \sigma_u)$ is a random intercept for study-site, $e_i \sim N(0,\sigma_e)$ is a residual variability around the linear prediction, for participant i. σ_u and σ_e are standard deviations for the random intercept and residual variability estimated in the model. For each combination of baseline haemoglobin values Hb0 between 8 and 17 g/dL and PQ actual dose of 0, 0.25, 0.4 mg/kg, age category and transmission intensity, 100,000 values of haemoglobin on Day 7 were simulated using equation (1), using the observed distribution of parasitaemia, and assuming G6PD deficiency. There was no correlation between baseline haemoglobin and baseline parasitaemia in our dataset, therefore the same distribution of parasitaemia was assumed for all baseline haemoglobin values. Proportion of participants with estimated haemoglobin on Day 7 below 7g/dl and 5g/dL thresholds were calculated for each combination of baseline haemoglobin and PQ dose, within all age categories and transmission intensity areas.

2. Shape of primaquine dose response relationship

Fractional polynomial analysis did not detect a nonlinear relationship between primaquine dose and drop in haemoglobin on Day 7 (p=0.292). Plots of the residuals for the linear model in patients with G6PD deficiency are shown below.

Relationship between residuals from the linear model and primaquine dose received. Line shows locally weighted regression smoothing.

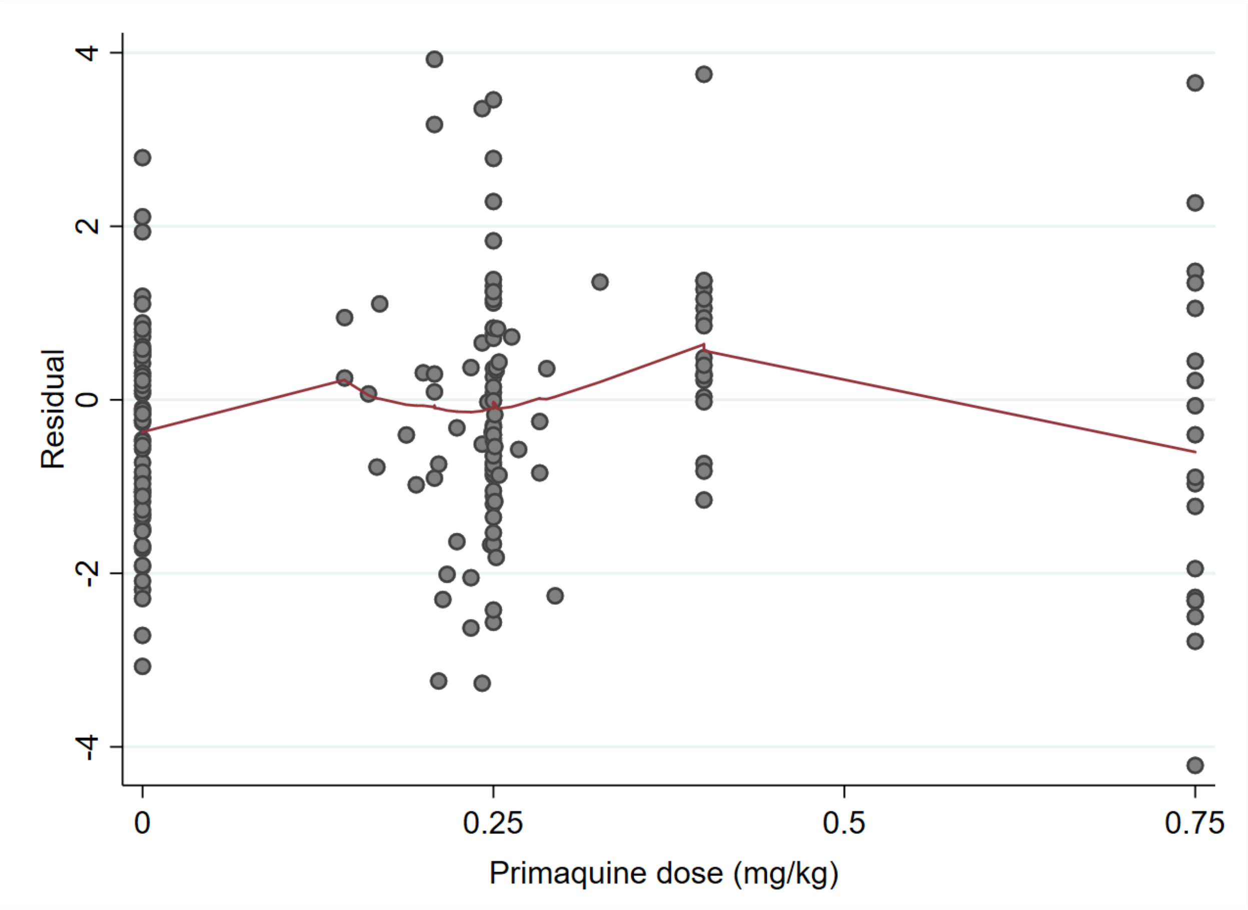

Observed versus fitted values (AC7) from the linear model shown by the primaquine target dose (mg/kg). Identity line (y=x) shown for reference.

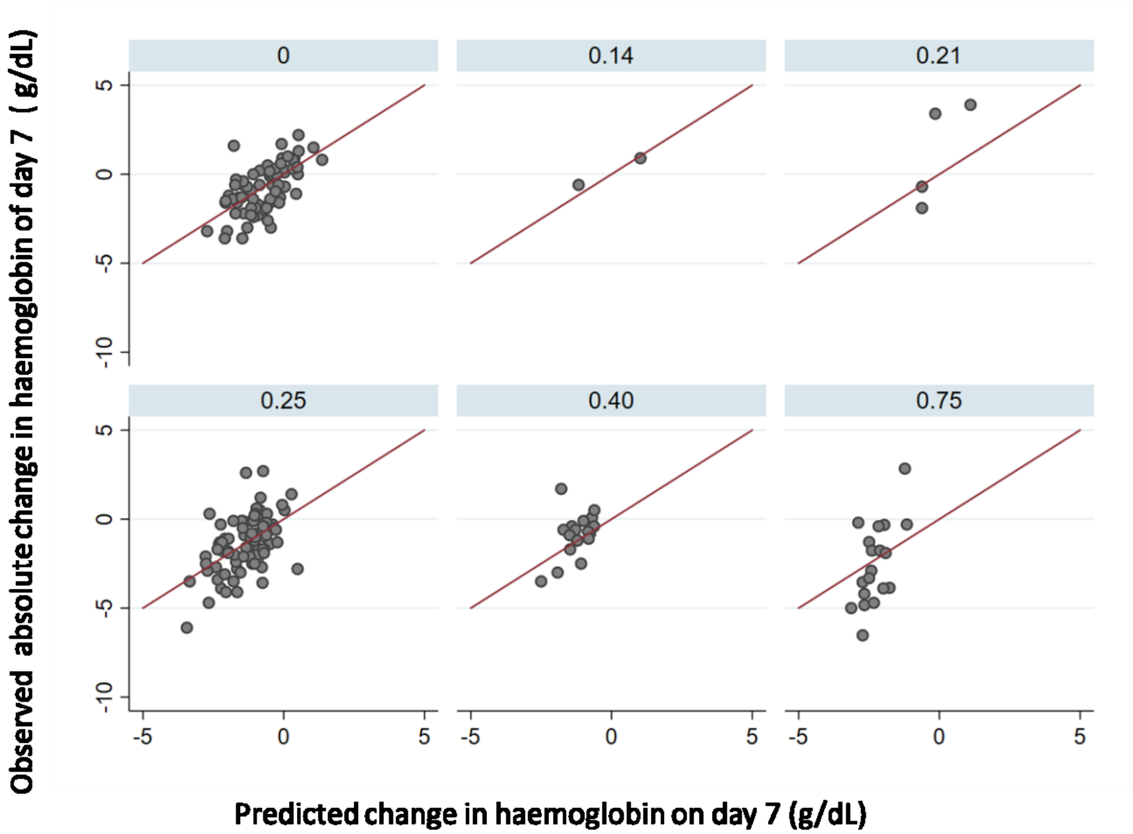

Comparison between the linear (red) and the best non-linear fractional polynomials (green) models

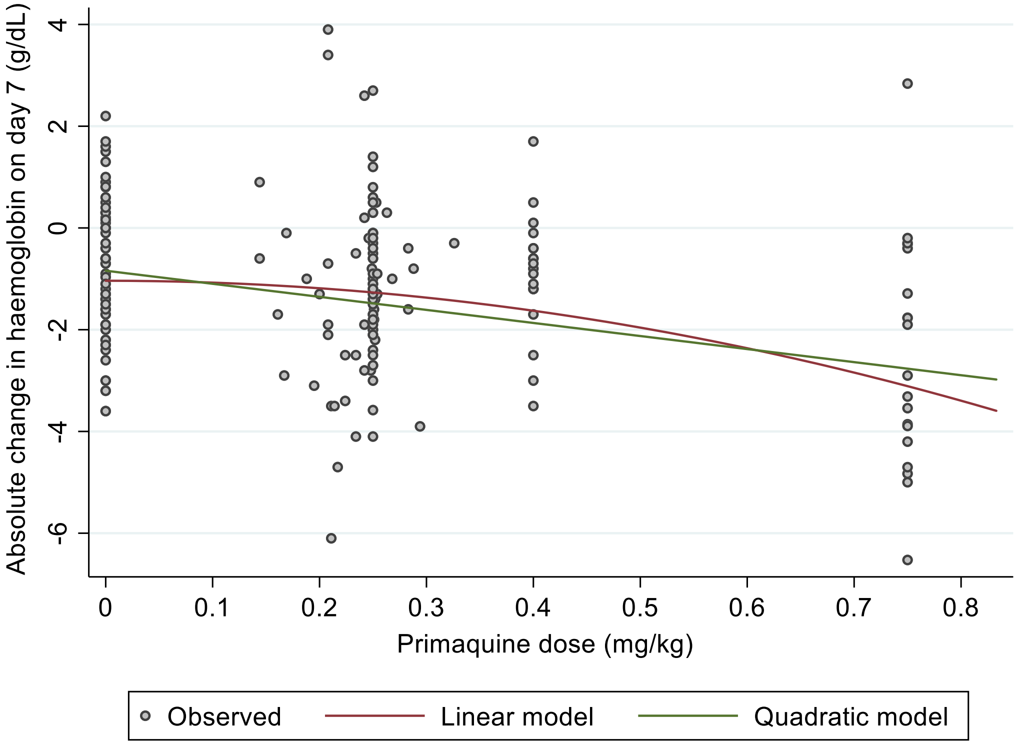

Supplement: Supplementary file 1 — Additional file 1:. Details of Methodology. [file 12916_2022_2504_MOESM1_ESM.pdf]
