# Supplementary material for: Safety of single-dose primaquine as a Plasmodium falciparum gametocytocide: a systematic review and meta-analysis of individual patient data
Source: BMC Med. 2022 Sep 16;20:350. doi: 10.1186/s12916-022-02504-z (PMC9479278; doi:10.1186/s12916-022-02504-z)
Supplement: Supplementary file 2 — Additional file 2: List L1. List of included studies. Table S1. Summary of characteristics of included studies. [file 12916_2022_2504_MOESM2_ESM.pdf]

List L1. List of included studies

1	Kaneko. A. An uncontrolled before and after study to examine the effect of a short-term mass drug administration (MDA) on malaria parasite prevalence on Ngodhe Island, Homa Bay County, Kenya [unpublished]
2	Okebe J, Bousema T, Affara M, Di Tanna GL, Dabira E, Gaye A, Sanya-Isijola F, Badji H, Correa S, Nwakanma D, Van Geertruyden JP, Drakeley C, D'Alessandro U. The Gametocytocidal Efficacy of Different Single Doses of Primaquine with Dihydroartemisinin-piperaquine in Asymptomatic Parasite Carriers in The Gambia: A Randomized Controlled Trial. EBioMedicine. 2016 Nov;13:348-355.
3	Mwaiswelo R, Ngasala BE, Jovel I, Gosling R, Premji Z, Poirot E, Mmbando BP, Björkman A, Mårtensson A. Safety of a single low-dose of primaquine in addition to standard artemether-lumefantrine regimen for treatment of acute uncomplicated Plasmodium falciparum malaria in Tanzania. Malar J. 2016 Jun 10;15:316.
4	Hamid MMA, Thriemer K, Elobied ME, Mahgoub NS, Boshara SA, Elsafi HMH, Gumaa SA, Hamid T, Abdelbagi H, Basheir HM, Marfurt J, Chen I, Gosling R, Price RN, Ley B. Low risk of recurrence following artesunate-Sulphadoxine-pyrimethamine plus primaquine for uncomplicated Plasmodium falciparum and Plasmodium vivax infections in the Republic of the Sudan. Malar J. 2018 Mar 16;17(1):117.
5	Gonçalves BP, Tiono AB, Ouédraogo A, Guelbéogo WM, Bradley J, Nebie I, Siaka D, Lanke K, Eziefula AC, Diarra A, Pett H, Bougouma EC, Sirima SB, Drakeley C, Bousema T.Single low dose primaquine to reduce gametocyte carriage and Plasmodium falciparum transmission after artemether-lumefantrine in children with asymptomatic infection: a randomised, double-blind, placebo-controlled trial. BMC Med. 2016 Mar 8;14:40.
6	El-Sayed B, El-Zaki SE, Babiker H, Gadalla N, Ageep T, Mansour F, Baraka O, Milligan P, Babiker A. A randomized open-label trial of artesunate- sulfadoxine-pyrimethamine with or without primaquine for elimination of sub-microscopic P. falciparum parasitaemia and gametocyte carriage in eastern Sudan. PLoS One. 2007 Dec 12;2(12):e1311
7	Samuels A, Kwambai T, Desai M, Kariuki S, Oneko M, Ochomo E, Otieno K, Smit M, Ter Kuile F, Gimnig J, ping Shi Y, Gosling R, Hwang J. Determining a safe and maximally efficacious dosing range of primaquine in combination with standard weight-based dosing of Dihydroartemisinin-piperaquine against Plasmodium falciparum gametocytes in G6PD-normal and -deficient patients with uncomplicated malaria. unpublished
8	Eziefula AC, Bousema T, Yeung S, Kamya M, Owaraganise A, Gabagaya G, Bradley J, Grignard L, Lanke KH, Wanzira H, Mpimbaza A, Nsobya S, White NJ, Webb EL, Staedke SG, Drakeley C. Single dose primaquine for clearance of Plasmodium falciparum gametocytes in children with uncomplicated malaria in Uganda: a randomised, controlled, double-blind, dose-ranging trial. Lancet Infect Dis. 2014 Feb;14(2):130-9.

9&10	Bastiaens GJH, Tiono AB, Okebe J, Pett HE, Coulibaly SA, Gonçalves BP, Affara M, Ouédraogo A, Bougouma EC, Sanou GS, Nébié I, Bradley J, Lanke KHW, Niemi M, Sirima SB, d'Alessandro U, Bousema T, Drakeley C. Safety of single low-dose primaquine in glucose-6-phosphate dehydrogenase deficient falciparum-infected African males: Two open-label, randomized, safety trials. PLoS One. 2018 Jan 11;13(1):e0190272.
11	Tine RC, Sylla K, Faye BT, Poirot E, Fall FB, Sow D, Wang D, Ndiaye M, Ndiaye JL, Faye B, Greenwood B, Gaye O, Milligan P. Safety and efficacy of adding a single low dose of primaquine to the treatment of adult patients with Plasmodium falciparum malaria in Senegal, to reduce gametocyte carriage: a randomized controlled trial. Clin Infect Dis. 2017 Aug 15;65(4):535-543.
12	Poirot E, Soble A, Ntshalintshali N, Mwandemele A, Mkhonta N, Malambe C, Vilakati S, Pan S, Darteh S, Maphalala G, Brown J, Hwang J, Pace C, Stergachis A, Vittinghoff E, Kunene S, Gosling R. Development of a pharmacovigilance safety monitoring tool for the rollout of single low-dose primaquine and artemether-lumefantrine to treat Plasmodium falciparum infections in Swaziland: a pilot study. Malar J. 2016 Jul 22;15(1):384.
13	Shekalaghe S, Drakeley C, Gosling R, Ndaro A, van Meegeren M, et al (2007) Primaquine Clears Submicroscopic Plasmodium falciparum Gametocytes that Persist after Treatment with Sulphadoxine-Pyrimethamine and Artesunate. PLoS ONE 2(10): e1023.
14	Shekalaghe SA, ter Braak R, Daou M, Kavishe R, van den Bijllaardt W, van den Bosch S, Koenderink JB, Luty AJ, Whitty CJ, Drakeley C, Sauerwein RW, Bousema T. In Tanzania, hemolysis after a single dose of primaquine coadministered with an artemisinin is not restricted to glucose-6-phosphate dehydrogenase-deficient (G6PD A-) individuals. Antimicrob Agents Chemother. 2010 May;54(5):1762-8.
15	Sutanto I, Suprijanto S, Kosasih A, Dahlan MS, Syafruddin D, Kusriastuti R, Hawley WA, Lobo NF, Ter Kuile FO. The effect of primaquine on gametocyte development and clearance in the treatment of uncomplicated falciparum malaria with dihydroartemisinin-piperaquine in South Sumatra, Western Indonesia: an open-label, randomized, controlled trial. Clin Infect Dis. 2013 Mar;56(5):685-93.
16	Raman J, Allen E, Workman L, Mabuza A, Swanepoel H, Malatje G, Frean J, Wiesner L, Barnes KI. Safety and tolerability of single low-dose primaquine in a low-intensity transmission area in South Africa: an open-label, randomized controlled trial. Malar J. 2019 Jun 24;18(1):209
17	Stone W, Sawa P, Lanke K, Rijpma S, Oriango R, Nyaurah M, Osodo P, Osoti V, Mahamar A, Diawara H, Woestenenk R, Graumans W, van de Vegte-Bolmer M, Bradley J, Chen I, Brown J, Siciliano G, Alano P, Gosling R, Dicko A, Drakeley C, Bousema T. A Molecular Assay to Quantify Male and Female Plasmodium falciparum Gametocytes: Results From 2 Randomized Controlled Trials Using Primaquine for Gametocyte Clearance. J Infect Dis. 2017 Aug 15;216(4):457-467.
18	Ley B, Alam MS, Thriemer K, Hossain MS, Kibria MG, Auburn S, Poirot E, Price RN, Khan WA. G6PD Deficiency and antimalarial efficacy for uncomplicated malaria in Bangladesh: a prospective observational study. PLoS One. 2016 Apr 29;11(4):e0154015.

19	Dicko A, Brown JM, Diawara H, Baber I, Mahamar A, Soumare HM, Sanogo K, Koita F, Keita S, Traore SF, Chen I, Poirot E, Hwang J, McCulloch C, Lanke K, Pett H, Niemi M, Nosten F, Bousema T, Gosling R. Primaquine to reduce transmission of Plasmodium falciparum malaria in Mali: a single-blind, dose-ranging, adaptive randomised phase 2 trial. Lancet Infect Dis. 2016 Jun;16(6):674-684. doi: 10.1016/S1473-3099(15)00479-X. Epub 2016 Feb 20
20	Bancone G, Chowwiwat N, Somsakchaicharoen R, Poodpanya L, Moo PK, Gornsawun G, Kajeechiwa L, Thwin MM, Rakthinthong S, Nosten S, Thinraow S, Nyo SN, Ling CL, Wiladphaingern J, Kiricharoen NL, Moore KA, White NJ, Nosten F. Single low dose primaquine (0.25 mg/kg) does not cause clinically significant haemolysis in G6PD-deficient subjects. PLoS One. 2016 Mar 24;11(3):e0151898.

Supplementary Table 1. Summary of characteristics of included studies

							Population at baseline					
Study ID	PubMed ID	Study Design	Location	Year	ACT	FU days	N	Malaria	Age	Pregnant	G6PD normal / deficient	Inclusion Hb cutoff (g/dL)
1	Unpublished	MDA	Kenya	2016	AP*	7	291	Either	All	Include	Either	≥ 7
2	27825738	RCT	The Gambia	2013-2015	DP	42	689	Pf asymptomatic	> 1 year	Exclude	Normal	≥ 8
3	27287612	RCT	Tanzania	2014	AL	28	220	Pf uncomplicated	≥ 1 year	Exclude	Either	≥ 8
4	29548285	RCT	Sudan	2015	ASSP	42	231	Pf uncomplicated	≥ 1 year	Exclude	Either**	≥ 8
5	26952094	RCT	Burkina Faso	2013-2014	AL	14	360	Pf asymptomatic	2 - 15 years	Not tested	Normal	≥ 8
6	18074034	RCT	Sudan	2004	ASSP	14	100	Pf asymptomatic	≥ 6 months	Exclude	Not tested	≥ 8
7	Unpublished	RCT	Kenya	2014-2015	DP	42	54	Pf uncomplicated	1 ≤ 12 years	Not tested	Both**	≥ 8
8	24239324	RCT	Uganda	2011	AL	28	454	Pf uncomplicated	1 - 10 years	Not tested	Normal	≥ 8
9	29324864	RCT & cohort	Burkina Faso	2014-2015	AL	28	78	Pf asymptomatic	18 - 45 years	Males only	Normal	≥ 11
10	29324864	RCT	The Gambia	2015-2016	DP	28	61	Pf asymptomatic	≥ 10 years	Males only	Either	≥ 11
11	28605472	RCT	Senegal	2014-2016	AL ASAQ DP	28	267	Pf uncomplicated	> 18 years	Exclude	Either	≥ 8
12	27450652	Cohort	eSwatini	2014-2015	AL	7	94	Pf uncomplicated	> 1 year	Exclude	Either	> 8
13	17925871	RCT	Tanzania	2006	ASSP	42	107	Pf uncomplicated	3 - 15 years	Not tested	Either	> 8
14	20194698	MDA	Tanzania	2008	ASSP	7	840	Not tested	1 - 12 years	Exclude	Either	> 8
15	23175563	RCT	Indonesia	2008-2010	DP	42	373	Pf uncomplicated	≥ 5 years	Exclude	Normal	≥ 8
16	31234865	RCT	South Africa	2016-2018	AL	42	140	Pf uncomplicated	> 1 year	Exclude	Either	≥ 7
17	28931236	RCT	Kenya	2014-2015	DP	14	114	Pf uncomplicated ***	5 - 15 years	Not tested	Not tested	> 9.5
18	27128675	Cohort	Bangladesh	2014-2015	AL	28	115	Pf uncomplicated	> 1 year	Males only	Either	≥ 8
19	26906747	RCT	Mali	2013-2014	DP	28	81	Pf uncomplicated ***	5 - 50 years	Males only	Normal	≥ 8
20	27010542	MDA	Thailand	2014	DP	7	1737	Not tested	> 6 mths	Exclude 1st trimester	Either	none

ACT = artemisinin-based combination therapy, AE = adverse event, AL = artemether-lumefantrine, ASAQ = artesunate-amodiaquine, ASSP = artesunate sulfadoxine-pyrimethamine, DP = dihydroartemisinin piperaquine, FST = fluorescent spot test, Hb = haemoglobin, MDA = mass drug administration, PCR = polymerase chain reaction, PQ = primaquine, RCT = randomised controlled trial *piperaquine if pregnant **normal if vivax ***gametocyte carriers

Supplementary Table 1. Summary of characteristics of included studies (continued)

	Methods		PQ treatment				Included in IPD meta-analysis		
Study ID	G6PD testing method used for IPD meta-analysis	Malaria diagnosis method	%	N	Day	Target dose	Hb	AE	Haemoglobinuria
1	Dojindo WST	Microscopy	98	286	0	0.144, 0.208	Y		
2	FST (Dimopolous)	RDT	75	515	2	0.2, 0.4, 0.75	Y	Y	Y
3	CareStart RDT (Access Bio)	Microscopy	50	110	0	0.25	Y	Y	Y
4	Carestart RDT (Access Bio)	Microscopy	52	119	2	0.25	Y		
5	FST	Microscopy	69	247	2	0.25, 0.4	Y	Y	y
6	Not applicable	Microscopy	49	49	3	0.75	Y		
7	FST (Trinity BioTech)	Microscopy	78	42	0	0.125, 0.25, 0.4, 0.75	Y	Y	y
8	FST (R&D Diagnostics)	Microscopy	75	339	2	0.1 0.4 0.75	Y		y
9	CareStart RDT (Access Bio)	Microscopy	87	68	0	0.25, 0.4	Y	Y	y
10	CareStart RDT (Access Bio)	Microscopy	82	50	0	0.25, 0.4	Y	Y	
11	CareStart RDT (Access Bio)	Microscopy	50	133	0	0.25	Y		y
12	CareStart RDT (Access Bio)	Not known	100	94	0	0.25	Y	Y	
13	PCR	Microscopy	50	54	2	0.75	Y		
14	PCR	Not known	93	784	2	0.75	Y		
15	FST (Trinity Biotech)	Microscopy	52	194	3	0.75	Y		
16	CareStart RDT (Access Bio)	RDT, microscopy	50	70	3	0.25	Y	Y	
17	Not applicable	Microscopy	51	58	2	0.25	Y		
18	FST (Randox UK)	Microscopy	100	115	2	0.75		Y	
19	Colorimetric quantification (R&D Diagnostics)	Microscopy	80	65	0	0.0625, 0.125, 0.25, 0.5	Y	Y	y
20	FST (R&D Diagnostics)	Not known	100	1,737	1	0.25		Y	

ACT = artemisinin-based combination therapy, AE = adverse event, AL = artemether-lumefantrine, ASAQ = artesunate-amodiaquine, ASSP = artesunate sulfadoxine-pyrimethamine, DP = dihydroartemisinin piperaquine, FST = fluorescent spot test, Hb = haemoglobin, MDA = mass drug administration, PCR = polymerase chain reaction PQ = primaquine, RCT = randomised controlled trial *piperaquine if pregnant **normal if vivax ***gametocyte carriers
