# Supplementary material for: Safety of single-dose primaquine as a Plasmodium falciparum gametocytocide: a systematic review and meta-analysis of individual patient data
Source: BMC Med. 2022 Sep 16;20:350. doi: 10.1186/s12916-022-02504-z (PMC9479278; doi:10.1186/s12916-022-02504-z)

Tables and Figures

Supplementary Table 2. Baseline characteristics of patients included in the analysis of haematological response. 2

Supplementary Table 3. Evaluation of variability in haemoglobin and association between haemoglobin change and baseline value. Only participants with normal G6PD status were included in this analysis. 4

Supplementary Table 4. Bi-variable analysis of factors associated with drop in haemoglobin on Day 7. 5

Supplementary Table 5. Bi-variable analysis of factors associated with anaemia on Day 7. 8

Supplementary Table 6. Univariable analysis of recovery from anaemia. 10

Supplementary Table 7. Haemoglobin measurements in patients who experienced severe anaemia at any time during the study. 12

Supplementary Table 8. Baseline characteristics of patients included in the analysis of adverse events 14

Supplementary Table 9. Listing of Serious Adverse Events reported in any study. 15

Supplementary Table 10. Baseline characteristics of patients included in the analysis of haemoglobinuria 17

Supplementary Table 11. Univariable analysis for haemoglobinuria 18

Supplementary Table 12. Multivariable model of absolute change in haemoglobin on Day 7 evaluated in studies from sub-Saharan Africa. 20

Supplementary Figure 1. Map of study sites 21

Supplementary Figure 2. Haemoglobin concentrations in G6PD deficient patients not included in the haematology analysis dataset. 22

Supplementary Figure 3. Relationship between haemoglobin at baseline and Day 7 as absolute change in patients (A) without G6PD deficiency and (B) with G6PD deficiency. 23

Supplementary Figure 4. Relationship between haemoglobin at baseline and Day 7 as fractional change in patients (A) without G6PD deficiency and (B) with G6PD deficiency. 24

Supplementary Figure 5. Relationship between baseline parasitaemia and haematological endpoints. 25

Supplementary Figure 6. Dose-response relationship for haematological endpoints. 26

Supplementary Figure 7. Time to recovery by day when anaemia was first recorded. 27

Supplementary Figure 8. Meta-analysis of odds of haemoglobinuria after PQ administration. 28

Supplementary Figure 9. Distribution of parasitaemia (observed) used in the simulation study. 29

Supplementary Figure 10. Predicted risk of severe anaemia on Day 7 (Hb<7g/dL) in Plasmodium falciparum patients with G6PD deficiency in sub-Saharan Africa. 30

Supplementary Figure 11. Predicted risk of very severe anaemia (Hb<5g/dL) on Day 7 in Plasmodium falciparum patients with G6PD deficiency in sub-Saharan Africa. 31

Supplementary Table 2. Baseline characteristics of patients included in the analysis of haematological response.

		PQ		no-PQ		All	
Sex: male		3260	1878 [57.6]	1259	715 [56.8]	4519	2593 [57.4]
Age (years)		3261	9.0 [1.0 - 91.0]	1259	11.0 [1.0 - 84.0]	4520	10.0 [1.0 - 91.0]
Age	< 5 years	3261	666 [20.4]	1259	209 [16.6]	4520	875 [19.4]
	5-11 years	3261	1339 [41.1]	1259	424 [33.7]	4520	1763 [39.0]
	12+ years	3261	1256 [38.5]	1259	626 [49.7]	4520	1882 [41.6]
WAZ score[1]		349	-0.6 [-3.5 - 2.9]	159	-0.6 [-3.8 - 2.5]	508	-0.6 [-3.8 - 2.9]
Underweight[1]		349	34 [9.7]	159	23 [14.5]	508	57 [11.2]
Malaria status	No	3261	747 [22.9]	1259	172 [13.7]	4520	919 [20.3]
	Pf	3261	1699 [52.1]	1259	1013 [80.5]	4520	2712 [60.0]
	Unknown	3261	815 [25.0]	1259	74 [5.9]	4520	889 [19.7]
G6PD status: deficient		2600	146 [5.6]	1092	74 [6.8]	3692	220 [6.0]
Pf parasite count (/µL)		1555	6020.0 [12.0 - 518180.0]	975	6700.0 [9.0 - 432000.0]	2530	6362.5 [9.0 - 518180.0]
Hyperparasiteamia[2]		2299	102 [4.4]	1134	35 [3.1]	3433	137 [4.0]
Temperature (C)		1885	36.7 [34.1 - 40.3]	1012	37.0 [34.0 - 40.5]	2897	36.8 [34.0 - 40.5]
Fever [3]		1886	405 [21.5]	1013	403 [39.8]	2899	808 [27.9]
Hb (g/dL)		3261	11.9 [6.0 - 20.1]	1259	11.9 [5.1 - 18.4]	4520	11.9 [5.1 - 20.1]
Severe Anaemia (Hb<7g/dL)		3261	1 [0.0]	1259	34 [2.7]	4520	35 [0.8]
Moderate Anaemia (Hb<10g/dL)		3261	433 [13.3]	1259	173 [13.7]	4520	606 [13.4]
Transmission Intensity [4]	Low	3261	2110 [64.7]	1259	884 [70.2]	4520	2994 [66.2]
	Moderate	3261	657 [20.1]	1259	200 [15.9]	4520	857 [19.0]
	High	3261	494 [15.1]	1259	175 [13.9]	4520	669 [14.8]
Region	Africa	3261	3067 [94.1]	1259	1080 [85.8]	4520	4147 [91.7]
	Asia	3261	194 [5.9]	1259	179 [14.2]	4520	373 [8.3]
ACT[5]	AL	3261	978 [30.0]	1259	466 [37.0]	4520	1444 [31.9]
	AP	3261	285 [8.7]	1259	5 [0.4]	4520	290 [6.4]

ASAQ	3261	45 [1.4]	1259	47 [3.7]	4520	92 [2.0]
ASSP	3261	996 [30.5]	1259	259 [20.6]	4520	1255 [27.8]
DP	3261	957 [29.3]	1259	482 [38.3]	4520	1439 [31.8]
PQ dose (mg/kg)	3261	0.4 [0.0 - 1.9]				
Target dose (mg/kg) <0.25	3261	612 [18.8]				
0.25	3261	803 [24.6]				
>0.25	3261	1846 [56.6]				
Day of PQ administration 0	3261	843 [25.9]				
1	3261	0 [0.0]				
2	3261	2109 [64.7]				
3	3261	309 [9.5]				

N, n [%] or N, median [range] shown

[1] evaluated in children < 5years of age, underweight is defined as waz score<-2; [2]defined as parasitaemia>100,000 parasites/µL; [3]defined as temperature>37.5C or history of fever; [4] Transmission Intensity areas defined based on estimates of P. falciparum prevalence rate (PfPR) [Gething 2011], assuming low transmission for study sites with a PfPR <0.15, moderate transmission if PfPR 0.15 to <0.40 and high transmission if PfPR ≥0.40; [5] AL =artemether-lumefnantrine, AP =artemisinin-piperaquine , ASAQ= artesunate-amodiaquine , ASSP= artesunate-sulfadoxine-pyrimethamine, DP=dihydroartemisinin-piperaquine.

Supplementary Table 3. Evaluation of variability in haemoglobin and association between haemoglobin change and baseline value.

Only participants with normal G6PD status were included in this analysis.

	Participants with Hb0<=9	Participants with Hb0>9
N	200	2995
SD0: Standard deviation of Hb0	0.623	1.691
SD7: standard deviation of Hb7	1.455	1.629
Mean Hb0	8.41	12.09
Mean Hb7	9.08	11.65
Coefficient of variation Hb0	0.074	0.140
Coefficient of variation Hb7	0.160	0.140
$r1$: Correlation coefficient (Hb0, Hb7)	0.371	0.633
$r2$: Correlation coefficient (Hb0, Hb7-Hb0) [1]	-0.062	-0.464
$r3$: Correlation coefficient (Hb0, (Hb7-Hb0)/Hb0)	-0.116	-0.404
$r4$: Correlation coefficient (Hb7-Hb0, (Hb0+Hb7)/2)[2]	**0.717**	**-0.050**
Correct ρ0 for testing correlation[3]	0.561	0.428
Z-statistics for testing $r2$= ρ_0[4]	8.027	-2.424
P-value	**<0.001**	**0.015**
Z-statistics for testing $r3$= ρ_0[5]	7.263	1.606
P-value	**<0.001**	**0.108**

[1]Please note that for any two series of independent random numbers x and y with the same standard deviation, one observes a strong correlation (1/√2≈ 0.71) between x-y and x.

[2] $r4 = -\frac{SD0^2 - SD7^2}{\sqrt{(SD0^2+SD7^2)^2 - 4r1SD0^2SD7^2}}$. If there is no difference in the variances of Hb0 and Hb7, the correlation r4 will be zero, i.e. change does not depend upon baseline value. If, on average, greater reduction can be obtained for greater baseline values, the post-treatment values will become 'closer' to each other, i.e. the variance of Hb7 will shrink and become smaller than that of Hb0. In other words, if there is a differential effect, this will manifest as a change of variances between the two measurements. As a result, if there is no difference in the variances between baseline and the follow-up measurement there is little evidence for the differential effect across the levels of baseline values.

[3] $\rho_0 = \sqrt{\frac{1-r1}{2}}$ is the corrected value of the null hypothesis for testing the correlation, derived assuming SD0=SD7.

[4] $Z = (z_r(r) - z_r(\rho_0)) / \sqrt{1/(n-3)}$, where z_r is a Fisher's z transformation $z_r(a) = \frac{1}{2} ln \frac{1+a}{1-a}$. r denotes $r2$ or $r3$, respectively. Positive values of Z-statistics represent a positive correlation between Hb0 and Hb7-Hb0 or (Hb7-Hb0)/Hb0), respectively.

Supplementary Table 4. Bi-variable analysis of factors associated with drop in haemoglobin on Day 7.

Estimates come from the normal or logistic regression model with random intercept for study site and are adjusted for baseline haemoglobin level.

	Absolute Change in Hb (g/dl)			Fractional drop >25%			
	N	change (95%CI)	P-value	n	%	OR (95% CI)	P-value
Sex							
Male	2211	0.20 (0.12, 0.28)	<0.001	60	2.7	0.68 (0.46, 1.02)	0.062
Female	1664	0.00		52	3.1		
Age (years)	3876	0.01 (0.00, 0.01)	<0.001	112	2.9	0.99 (0.97, 1.01)	0.242
Age category							
< 5 years	733	-0.57 (-0.72, -0.41)	<0.001	27	3.7	3.23 (1.51, 6.91)	0.003
5-11 years	1561	-0.30 (-0.41, -0.19)	<0.001	35	2.2	1.35 (0.73, 2.50)	0.341
12+ years	1581	0.00		50	3.2	1.00	
WAZ score[1]	476	0.03 (-0.06, 0.11)	0.534	8	1.7	0.74 (0.36, 1.50)	0.402
Nutritional status[1]							
Underweight	426	-0.26 (-0.57, 0.04)	0.088	7	1.6	1.83 (0.19, 17.16)	0.597
Normal	50	0.00		1	2.0	1.00	
G6PD status							
Deficient	194	-0.59 (-0.79, -0.38)	<0.001	20	10.4	5.67 (2.99, 10.75)	<0.001
Normal	3170	0.00		78	2.6	1.00	
Log10 parasitaemia	3250	-0.15 (-0.20, -0.10)	<0.001	77	2.4	1.29 (1.06, 1.58)	0.013
Hyperparasiteamia[2]							
No detectable parasitaemia	929	0.00		15	1.6	1.00	
Parasitaemia<100,000 /µL	2184	0.05 (-0.11, 0.22)	0.519	59	2.7	1.16 (0.49, 2.73)	0.737
Parasitaemia>100,000 / µL	137	-0.72 (-0.99, -0.45)	<0.001	3	2.2	2.02 (0.45, 9.09)	0.359
Temperature (C)	2556	-0.06 (-0.12, 0.00)	0.057	66	2.6	1.22 (0.96, 1.56)	0.105
Fever[3]							

Yes	694	-0.22 (-0.38, -0.06)	0.006	34	4.9	2.11 (1.15, 3.86)	0.016
No	1864	0.00		32	1.7		
Transmission intensity[4]							
Low	2444	0.00		96	3.9	1.00	
Moderate	769	0.06 (-0.47, 0.59)	0.824	11	1.4	0.45 (0.17, 1.19)	0.109
High	662	0.29 (-0.26, 0.84)	0.304	5	0.8	0.24 (0.06, 0.99)	0.048
Region							
Africa	3510	0.27 (-0.87, 1.42)	0.642	89	2.5	0.40 (0.07, 2.36)	0.314
Asia	365	0.00		23	6.3	1.00	
PQ administration							
Yes	2781	-0.01 (-0.11, 0.08)	0.753	87	3.1	1.45 (0.88, 2.40)	0.145
No	1095	0.00		25	2.3	1.00	
PQ dose (0.1mg/kg)	3876	-0.12 (-0.29, 0.04)	0.149	112	2.9	2.82 (1.24, 6.40)	0.013
Target dose							
0	1094	0.00		25	2.3	1.00	
Low	531	0.04 (-0.13, 0.21)	0.683	5	0.9	0.57 (0.16, 2.03)	0.385
0.25	692	0.04 (-0.09, 0.18)	0.528	18	2.6	1.20 (0.61, 2.38)	0.595
High	1558	-0.07 (-0.18, 0.04)	0.222	64	4.1	1.80 (1.00, 3.22)	0.049
Time of PQ							
no PQ	1094	0.00		25	2.3	1.00	
day 0	702	-0.03 (-0.21, 0.15)	0.755	17	2.4	0.91 (0.42, 1.95)	0.804
day 2	1811	0.04 (-0.08, 0.16)	0.546	52	2.9	1.86 (0.80, 4.32)	0.148
day 3	268	-0.15 (-0.36, 0.05)	0.146	18	6.7	1.82 (0.86, 3.85)	0.117
ACT[5]							
AL	1321	-0.07 (-0.40, 0.25)	0.659	23	1.7	1.03 (0.40, 2.64)	0.948
AP	227	0.73 (-0.41, 1.87)	0.207	3	1.3	0.45 (0.06, 3.26)	0.431
ASAQ	77	-0.20 (-0.58, 0.18)	0.306	8	10.4	3.48 (1.03, 11.74)	0.044
ASSP	929	0.29 (-0.28, 0.86)	0.320	42	4.5	1.69 (0.56, 5.09)	0.349
DP	1321	0.00		36	2.7	1.00	

[1] evaluated in children < 5years of age, underweight is defined as waz score<-2; [2]defined as parasitaemia>100,000 parasites/µL; [3]defined as temperature>37.5C or history of fever; [4] TIA=Transmission Intensity Areas defined based on estimates of P. falciparum prevalence rate (PfPR), assuming low transmission for

study sites with a PfPR <0.15, moderate transmission if PfPR 0.15 to <0.40 and high transmission if PfPR ≥0.40; [5] AL =artemether-lumefnantrine, AP =artemisinin-piperaquine , ASAQ= artesunate-amodiaquine , ASSP= artesunate-sulfadoxine-pyrimethamine, DP=dihydroartemisinin-piperaquine.

Supplementary Table 5. Bi-variable analysis of factors associated with anaemia on Day 7.

Estimates come from the logistic regression model with random intercept for study site and are adjusted for baseline haemoglobin level.

		Severe or Moderate Anaemia				Severe Anaemia			
	N	n	%	OR (95% CI)	P-value	n	%	OR (95% CI)	P-value
Sex									
Male	2212	339	15.3	1.08 (0.87, 1.33)	0.478	15	0.7	0.81 (0.38, 1.73)	0.576
Female	1664	300	18.0	1.00		20	1.2	1.00	
Age	3875	639	16.5	0.99 (0.98, 1.00)	0.054	35	0.9	1.00 (0.96, 1.05)	0.949
Age category									
< 5 years	733	294	40.1	3.17 (2.16, 4.65)	<0.001	17	2.3	1.07 (0.25, 4.52)	0.931
5-11 years	1561	234	15.0	1.65 (1.18, 2.30)	0.003	11	0.7	1.14 (0.30, 4.37)	0.846
12+ years	1581	111	7.0	1.00		7	0.4	1.00	
WAZ score[1]	476	176	37.0	1.06 (0.86, 1.30)	0.617	4	0.8	1.30 (0.37, 4.55)	0.686
Nutritional status[1]									
Underweight	426	26	52.0	1.77 (0.87, 3.60)	0.113	3	0.7	5.56 (0.05, 563.13)	0.466
Normal	50	150	35.2	1.00		1	2.0	1.00	
G6PDS status									
Deficient	194	44	22.7	3.41 (2.02, 5.78)	<0.001	7	3.6	13.67 (4.25, 43.95)	<0.001
Normal	3170	533	16.8	1.00		17	0.6	1.00	
Log10 parasitaemia	3250	480	14.8	1.27 (1.11, 1.45)	<0.001	15	0.5	0.81 (0.53, 1.22)	0.313
Hyperparasiteamia[2]									
No detectable parasitaemia	929	120	12.9	1.00		8	0.9	1.00	
Parasitaemia<100,000 /µL	2184	305	14.0	0.96 (0.61, 1.53)	0.878	5	0.2	0.38 (0.07, 2.24)	0.287
Parasitaemia>100,000 / µL	137	55	40.1	3.36 (1.74, 6.49)	<0.001	2	1.5	1.95 (0.17, 22.08)	0.589
Temperature (C)	2556	325	12.7	1.08 (0.92, 1.27)	0.348	14	0.5	0.88 (0.47, 1.67)	0.695
Fever[3]									
Yes	694	110	15.9	1.48 (0.96, 2.28)	0.075	3	0.4	0.69 (0.14, 3.47)	0.657
No	1864	215	11.5	1.00		11	0.6	1.00	

Transmission Intensity[4]									
Low	2444	423	17.3	1.00		27	1.1	1.00	
Moderate	769	97	12.6	1.09 (0.42, 2.82)	0.864	8	1.0	1.23 (0.28, 5.41)	0.782
High	662	119	18.0	0.78 (0.27, 2.27)	0.648	0		ND	
Region									
Africa	3510	599	17.1	0.54 (0.08, 3.74)	0.532	35	1.0	ND	
Asia	365	40	11.0	1.00		0		1.00	
PQ administration									
Yes	2781	483	17.4	1.51 (1.16, 1.96)	0.002	22	0.8	1.35 (0.53, 3.44)	0.531
No	1094	156	14.3	1.00		13	1.3	1.00	
PQ dose (0.1mg/kg)	3875	639	16.5	1.82 (1.18, 2.82)	0.007	35	0.9	3.53 (0.83, 15.07)	0.089
Target dose									
0	1095	156	14.3	1.00		13	1.2	1.00	
Low	531	85	16.0	1.63 (1.07, 2.49)	0.024	3	0.6	0.68 (0.10, 4.75)	0.698
0.25	692	101	14.6	1.47 (0.99, 2.20)	0.058	2	0.3	0.56 (0.09, 3.27)	0.516
High	1558	297	19.1	1.49 (1.09, 2.05)	0.013	17	1.1	2.11 (0.73, 6.16)	0.170
Time of PQ									
no PQ	1094	156	14.3	1.00		13	1.2	1.00	
day 0	702	114	16.2	1.54 (0.96, 2.47)	0.073	4	0.6	0.55 (0.10, 2.95)	0.486
day 2	1811	334	18.4	1.36 (0.97, 1.90)	0.071	16	0.9	1.64 (0.54, 4.98)	0.385
day 3	268	35	13.1	2.19 (1.15, 4.15)	0.016	2	0.7	7.38 (0.63, 86.93)	0.112
ACT[5]									
AL	1321	237	17.9	1.35 (0.58, 3.16)	0.484	4	0.3	1.52 (0.28, 8.36)	0.631
AP	227	21	9.3	1.19 (0.16, 8.73)	0.866	6	2.6	20.60 (3.79, 111.88)	<0.001
ASAQ	77	9	11.7	2.44 (0.77, 7.79)	0.131	0		ND	
ASSP	939	198	21.3	0.54 (0.17, 1.66)	0.280	23	2.5	8.41 (1.93, 36.73)	0.005
DP	1321	174	13.2	1.00		2	0.2	1.00	

[1] evaluated in children < 5years of age, underweight is defined as waz score<-2; [2]defined as parasitaemia>100,000 parasites/µL; [3]defined as temperature>37.5C or history of fever; [4] Transmission Intensity Areas defined based on estimates of P. falciparum prevalence rate (PfPR), assuming low transmission for study sites with a PfPR <0.15, moderate transmission if PfPR 0.15 to <0.40 and high transmission if PfPR ≥0.40; ; [5] AL =artemether-lumefnantrine, AP =artemisinin-piperaquine , ASAQ= artesunate-amodiaquine , ASSP= artesunate-sulfadoxine-pyrimethamine, DP=dihydroartemisinin-piperaquine.

Supplementary Table 6. Univariable analysis of recovery from anaemia.

	N	n	HR (95% CI)	P-value	PH p-value[6]
Sex					
Male	366	318	0.99 (0.84, 1.16)	0.904	0.942
Female	342	282			
Age	708	600	1.00 (1.00, 1.01)	0.258	0.871
Age category					
< 5 years	268	216	0.68 (0.52, 0.88)	0.004	0.320
5-11 years	288	258	1.04 (0.82, 1.33)	0.735	
12+ years	152	126	1.00		
WAZ score[1]	263	212	1.05 (0.92, 1.19)	0.495	0.908
Nutritional status[1]					
Underweight	39	28	0.89 (0.59, 1.35)	0.591	0.298
Normal	224	184	1.00		
G6PD status					
Deficient	41	36	0.81 (0.56, 1.16)	0.250	0.050
Normal	633	547	1.00		
Log10 parasitaemia	706	599	1.00 (0.93, 1.08)	0.917	<0.001
Hyperparasitaemia[2]					
Parasitaemia<100,000 /µL	623	526	1.00		
Parasitaemia>100,000 / µL	75	66	0.74 (0.56, 0.98)	0.033	0.023
Temperature (C)	464	378	1.01 (0.90, 1.13)	0.925	0.003
Fever[3]					
Yes	138	125	1.12 (0.82, 1.52)	0.472	<0.001
No	326	253	1.00		
TIA[4]					
Low	355	308	1.00		0.227
Moderate	131	90	0.74 (0.49, 1.10)	0.139	
High	222	202	1.04 (0.67, 1.61)	0.863	

Region					
Africa	708	600	ND		
Asia	0		ND		
PQ administration					
Yes	708	600	1.02 (0.86, 1.22)	0.812	0.090
No			1.00		
PQ dose (0.1mg/kg)	708	600	1.17 (0.86, 1.59)	0.324	0.467
Target dose					
0	226	185	1.00		0.002
Low	124	103	0.94 (0.73, 1.23)	0.666	
0.25	113	93	0.93 (0.71, 1.23)	0.616	
High	245	219	1.13 (0.91, 1.41)	0.264	
Time of PQ					
no PQ	226	185	1.00		0.001
day 0	130	108	0.94 (0.72, 1.24)	0.681	
day 2	342	300	1.08 (0.88, 1.34)	0.464	
day 3	10	7	0.65 (0.28, 1.50)	0.314	
ACT[5]					
AL	351	306	0.90 (0.56, 1.43)	0.648	<0.001
AP	23	9	0.79 (0.30, 2.08)	0.634	
ASAQ	17	17	1.05 (0.54, 2.05)	0.889	
ASSP	66	59	1.62 (0.85, 3.10)	0.146	
DP	251	209	1.00		

N =number evaluated, n= number who cleared anaemia

[1] evaluated in children < 5years of age, underweight is defined as waz score<-2; [2]defined as parasitaemia>100,000 parasites/µL; [3]defined as temperature>37.5C or history of fever; [4] TIA=Transmission Intensity Areas defined based on estimates of P. falciparum prevalence rate (PfPR) assuming low transmission for study sites with a PfPR <0.15, moderate transmission if PfPR 0.15 to <0.40 and high transmission if PfPR ≥0.40; [5] AL =artemether-lumefnantrine, AP =artemisinin-piperaquine , ASAQ= artesunate-amodiaquine , ASSP= artesunate-sulfadoxine-pyrimethamine, DP=dihydroartemisinin-piperaquine; [6]PH p-value =p-value for the test of the proportional hazards assumption based on Schoenfeld residuals

Supplementary Table 7. Haemoglobin measurements in patients who experienced severe anaemia at any time during the study.

Another 17 participants (all no primaquine group) had severe anaemia at baseline and no further data.

Haemoglobin measurements (g/dL)							PQ dose (mg/kg)	G6PD status	Age (years)
Day 0	Day 2	Day 3	Day 7	Day 14	Day 21	Day 28			
Severe Anaemia at baseline									
6.9			7.1				0		2
6.923		7.567	7.567	9.821		11.431	0	normal	3
6.762		6.923	6.601	8.533		10.143	0	normal	3
6.966			6.2				0	normal	4
5.3			4.9				0	normal	4
6.7			7.8				0	normal	4
6.7	6.8		5.5				0		29
5.508			6.3				0		2
6.48			7.2				0		2
6.3			7				0	normal	5
5.9			7				0		8
6.156			10				0		5
6.4			5.6				0		4
6	6.1		6.4				0		22
6.318			5.4				0	normal	2
6.156			8				0	normal	1
6.48			7.9				0	normal	1
6		11.7	12.2				0.25	normal	10
Severe anaemia day 2-3									
8.2	6.2	8.7	8.1	7.2	7.1	8.2	0	normal	4
7.1	6		5.3				0	normal	10
8.4	6.7	7.4	8	7.9	8.1	9.6	0.1	normal	6
7.4	6.6						0.144		20
7.1	6.4		6.2				0.144		26
8.3	6.7						0.144		37
9.5	6.8	8.8			8		0.4	normal	3
9	6.6	7.1	8.8	10.2	11.9	10.4	0.75	normal	2.58
8.9	6.3	7.4	8.5	10	10.5	11.9	0.75	normal	2.5
8.3	6.6	4.7	6.6	10.2	12.9	11.8	0.75	normal	7
8.8		6.8	6	8.7	9.9	10.9	0	normal	2
8.1		6	6.6	9.8	10.5	10.9	0	deficient	1
8.7		6.8	8.6	8.7	9.1	11.7	0	deficient	23
8.3	7.2	6.2	8.1	10.1	10	10	0.1	normal	1.5

8.2		6.3	6.8	8.2	10	11.1	0.25	deficient	4
9.66		6.44	8.05	7.728		9.016	0.75	normal	2
Severe anaemia on Day 7									
7.5			6.4				0	normal	2
14.3	12.5	10.1	6.8	9.9	11.9		0	normal	14
15.5	14.6		6.7				0.144		53
8.3	7.1		6.7				0.144		32
8.8		7.7	6	7		7.1	0.25	deficient	37.7796
11.826			5.3				0.75	deficient	2
11.6			5.2				0.75	normal	1
11.016			6.2				0.75	normal	7
7.889		7.245	5.474	8.694		10.948	0.75	normal	9
8.2			6.8				0.75	normal	6
8.7			5	8.3			0.75		4
9			6.5				0.75	normal	6
9.1			6.966				0.75	normal	4
11.1			6.6				0.75		1
8.1			5.832				0.75	normal	1
9.558			5.7				0.75	deficient	2
8.9			6.9				0.75		5
11.502			6.8				0.75	deficient	5
10.692			6.8				0.75	deficient	5
8.2			6				0.75	deficient	6
8.3			4.8				0.75		5
Severe anaemia after Day 7									
11.2	9.1	9.5	8.3	6.7	9.1	11.1	0.1	normal	2.25
9.9	9	8.9	8.5	4.9	9.2	10.6	0.4	normal	1.5
8.2		7	7	7.3	4.5		0.25	normal	1
7.728		8.372	7.406	8.372		6.923	0	normal	3
10.5		10.4	11	10.8	10	6.6	0	normal	2

Supplementary Table 8. Baseline characteristics of patients included in the analysis of adverse events

		PQ		no-PQ		All	
Sex: male		3111	1811 [58.2]	516	298 [57.8]	3627	2109 [58.1]
Age		3113	12.0 [0.5 - 75.0]	516	11.0 [1.0 - 84.0]	3629	11.0 [0.5 - 84.0]
Age	< 5 years	3113	482 [15.5]	516	76 [14.7]	3629	558 [15.4]
	5-11 years	3113	1059 [34.0]	516	208 [40.3]	3629	1267 [34.9]
	12+ years	3113	1572 [50.5]	516	232 [45.0]	3629	1804 [49.7]
WAZ score		477	-0.5 [-5.8 - 4.0]	76	-0.5 [-3.5 - 2.5]	553	-0.5 [-5.8 - 4.0]
Underweight (waz score<-2)		477	58 [12.2]	76	8 [10.5]	553	66 [11.9]
Pf Malaria status	No	3113	1171 [37.6]	516	145 [28.1]	3629	1316 [36.3]
	Yes	3113	895 [28.8]	516	355 [68.8]	3629	1250 [34.4]
	Unknown	3113	1047 [33.6]	516	16 [3.1]	3629	1063 [29.3]
G6PD status: deficient		3089	255 [8.3]	511	46 [9.0]	3600	301 [8.4]
Pf parasite count (/µL)		796	1643.5 [12.0 - 1.3e+06]	350	2067.5 [9.0 - 386800.0]	1146	1789.5 [9.0 - 1.3e+06]
Hyperparasitaemia[1]		1235	29 [2.3]	489	12 [2.5]	1724	41 [2.4]
Temperature (C)		3029	36.9 [34.4 - 40.3]	617	36.8 [35.0 - 40.4]	3646	36.9 [34.4 - 40.4]
Fever[2]		2897	36.9 [34.4 - 40.3]	483	36.6 [35.6 - 40.4]	3380	36.9 [34.4 - 40.4]
Hb (g/dL)		1376	11.8 [6.0 - 20.8]	511	11.7 [8.0 - 17.8]	1887	11.8 [6.0 - 20.8]
Severe Anaemia (Hb<7g/dL)		1376	1202 [87.4]	511	436 [85.3]	1887	1638 [86.8]
Moderate Anaemia (Hb<10g/dL)		1376	1 [0.1]	511	0 [0.0]	1887	1 [0.1]
Transmission Intensity [2]	Low	3113	2691 [86.4]	516	365 [70.7]	3629	3056 [84.2]
	Moderate	3113	267 [8.6]	516	91 [17.6]	3629	358 [9.9]
	High	3113	155 [5.0]	516	60 [11.6]	3629	215 [5.9]
Region	Africa	3113	1261 [40.5]	516	516 [100.0]	3629	1777 [49.0]
	Asia	3113	1852 [59.5]	516	0 [0.0]	3629	1852 [51.0]

[1] defined as parasitaemia>100,000 parasites/µL, [2] defined as temperature>37.5 or history of fever; [3] TIA=Transmission Intensity Areas defined based on estimates of P. falciparum prevalence rate (PfPR) [Gething 2011], assuming low transmission for study sites with a PfPR <0.15, moderate transmission if PfPR 0.15 to <0.40 and high transmission if PfPR ≥0.40;

Supplementary Table 9. Listing of Serious Adverse Events reported in any study.

Study ID	Patient ID	PQ[1]	ACT Day 0[2]	Age in years	G6PD status	Dose PQ[3]	PQ dosing day4	AE system organ class	AE preferred term	Day AE started[5]	Relatedness[6]	Outcome
**Up to Day 7**												
3	5	N	AL	23	Deficient	0	Day 0	Blood and lymphatic system disorders	Haemolytic anaemia	2	Not known	Recovered / resolved
3	6	Y	AL	3	Normal	0.25	Day 0	Blood and lymphatic system disorders	Haemolytic anaemia	1	Not known	Recovered / resolved
3	7	N	AL	2	Normal	0	Day 0	Blood and lymphatic system disorders	Anaemia	2	Not known	Recovered / resolved
3	8	Y	AL	4	Deficient	0.25	Day 0	Blood and lymphatic system disorders	Anaemia	1	Not known	Recovered / resolved
3	9	N	AL	1	Deficient	0	Day 0	Blood and lymphatic system disorders	Haemolytic anaemia	1	Not known	Recovered / resolved
3	10	Y	AL	30	Normal	0.25	Day 0	Blood and lymphatic system disorders	Haemolytic anaemia	1	Not known	Recovered / resolved
7	11	Y	DP	4	Normal	0.125	Day 0	Gastrointestinal disorders	Vomiting	0	Possible	Recovered / resolved
12	20[7]	Y	AL	31	Normal	0.187	Day 0	Gastrointestinal disorders	Diarrhoea	1	Not related	Recovered / resolved
12	20[7]	Y	AL	31	Normal	0.187	Day 0	Gastrointestinal disorders	Vomiting	1	Not related	Recovered / resolved
12	22[7,8]	Y	AL	60	Normal	0.221	Day 0	Gastrointestinal disorders	Diarrhoea	-1	Not related	Fatal
12	22[7,8]	Y	AL	60	Normal	0.221	Day 0	Gastrointestinal disorders	Vomiting	-1	Not related	Fatal
7	12	Y	DP	3	Normal	0.75	Day 0	Investigations	Haemoglobin decreased	7	Possible	Recovered / resolved
7	14	Y	DP	5	Normal	0.75	Day 0	Investigations	Haemoglobin decreased	7	Unlikely	Recovered / resolved
7	15	Y	DP	11	Normal	0.4	Day 0	Investigations	Haemoglobin decreased	3	Possible	Recovered / resolved
7	16	Y	DP	2	Normal	0.25	Day 0	Investigations	Haemoglobin decreased	1	Possible	Recovered / resolved
7	17	N	DP	4	Normal	0	Day 0	Investigations	Haemoglobin decreased	3	Possible	Recovered / resolved
7	18	Y	DP	2	Deficient	0.25	Day 0	Investigations	Haemoglobin decreased	1	Possible	Recovered / resolved
2	3	Y	DP	4	Normal	0.2	Day 2	General disorders and administration site conditions	Injection site injury	3	Not related	Recovered / resolved

12	22[7,8]	Y	AL	60	Normal	0.221	Day 0	General disorders and administration site conditions	Pyrexia	-1	Not related	Fatal
12	22[7,8]	Y	AL	60	Normal	0.221	Day 0	General disorders and administration site conditions	Chills	-1	Not related	Fatal
12	21[8]	Y	AL	28	Normal	0.231	Day 0	Infections and infestations	Malaria	-2	Not related	Fatal
12	20[7]	Y	AL	31	Normal	0.187	Day 0	Nervous system disorders	Headache	1	Not related	Recovered / resolved
12	20[7]	Y	AL	31	Normal	0.187	Day 0	Nervous system disorders	Dizziness	1	Not related	Recovered / resolved
**Day 8 to 28**												
2	1	Y	DP	11	Normal	0.75	Day 2	Infections and infestations	Pneumonia	11	Not related	Recovered / resolved
2	2	Y	DP	8	Normal	0.2	Day 2	Infections and infestations	Pneumonia	10	Unlikely	Recovered / resolved
7	13	Y	DP	4	Normal	0.25	Day 0	Infections and infestations	Pneumonia	27	Not related	Recovered / resolved
20	24	Y	DP	35	Normal	0.25	Day 1	Infections and infestations	HIV infection	16	Not related	Not known
20	24	Y	DP	35	Normal	0.25	Day 1	Infections and infestations	Tuberculosis	16	Not related	Not known
2	4	Y	DP	Not known	Normal	Not known	Day 2	Injury, poisoning and procedural complications	Injury	Not known	Not related	Recovered / resolved
16	23	N	AL	43	Normal	0	Day 3	Renal and urinary disorders	Renal impairment	9	Not related	Recovered / resolved
7	19	Y	DP	2	Normal	0.75	Day 0	Respiratory, thoracic and mediastinal disorders	Asthma	15	Not related	Recovered / resolved
20	24	Y	DP	36	Normal	0.25	Day 1	Renal and urinary disorders	Haemoglobinuria	Not known	Not known	Not known

[1] was PQ (primaquine) administered, Y=yes, N=no; [2] ACT=artemisinin-based combination therapy, DP = dihydroartemisinin-piperaquine, AL = artemether-lumefantrine; [3] Actual dose (mg/kg); [4] day of PQ administration measured from day 0 when ACT was administered; [5] day AE was observed , measured since the PQ administration; [6] assessment according to primary study authors; [7] several symptoms of undiagnosed SAE; [8] data included as symptoms started before PQ dose, then worsened after dose

Supplementary Table 10. Baseline characteristics of patients included in the analysis of haemoglobinuria

		PQ		no-PQ		All	
Sex: male		1507	897 [59.5]	677	382 [56.4]	2184	1279 [58.6]
Age (years)		1507	9.0 [1.0 - 75.0]	677	10.0 [1.0 - 84.0]	2184	9.0 [1.0 - 84.0]
Age	< 5 years	1507	309 [20.5]	677	130 [19.2]	2184	439 [20.1]
	5-11 years	1507	688 [45.7]	677	260 [38.4]	2184	948 [43.4]
	12+ years	1507	510 [33.8]	677	287 [42.4]	2184	797 [36.5]
WAZ score		304	-0.6 [-3.5 - 2.6]	129	-0.5 [-3.5 - 2.5]	433	-0.5 [-3.5 - 2.6]
Underweight (waz score<-2)		304	28 [9.2]	129	15 [11.6]	433	43 [9.9]
Pf Malaria status	No	1507	393 [26.1]	677	130 [19.2]	2184	523 [23.9]
	Yes	1507	1077 [71.5]	677	529 [78.1]	2184	1606 [73.5]
	Unknown	1507	37 [2.5]	677	18 [2.7]	2184	55 [2.5]
G6PD status: deficient		1504	88 [5.9]	675	53 [7.9]	2179	141 [6.5]
Pf parasite count (/µL)		1470	958.5 [0.0 - 518180.0]	659	2760.0 [0.0 - 432000.0]	2129	1480.0 [0.0 - 518180.0]
Hyperparasiteamia[1]		1470	99 [6.7]	659	34 [5.2]	2129	133 [6.2]
Hb (g/dL)		1504	11.6 [6.0 - 18.2]	673	11.8 [8.0 - 18.4]	2177	11.7 [6.0 - 18.4]
No anaemia		1504	1290 [85.8]	673	575 [85.4]	2177	1865 [85.7]
Moderate anaemia		1504	213 [14.2]	673	98 [14.6]	2177	311 [14.3]
Severe anaemia		1504	1 [0.1]	673	0 [0.0]	2177	1 [0.0]
Malaria treatment	AL	1507	809 [53.7]	677	396 [58.5]	2184	1205 [55.2]
	DP	1507	45 [3.0]	677	45 [6.6]	2184	90 [4.1]
	ASAQ	1507	653 [43.3]	677	236 [34.9]	2184	889 [40.7]
Transmission Intensity [2]	Low	1507	756 [50.2]	677	415 [61.3]	2184	1171 [53.6]
	Moderate	1507	258 [17.1]	677	89 [13.1]	2184	347 [15.9]
	High	1507	493 [32.7]	677	173 [25.6]	2184	666 [30.5]

[1] defined as parasitaemia>100,000 parasites/µL, [2] TIA=Transmission Intensity Areas defined based on estimates of P. falciparum prevalence rate (PfPR) [Gething 2011], assuming low transmission for study sites with a PfPR <0.15, moderate transmission if PfPR 0.15 to <0.40 and high transmission if PfPR ≥0.40.

Supplementary Table 11. Univariable analysis for haemoglobinuria

	N	n	OR (95% CI)	P-value
Sex				
Male	1279	34	0.82 (0.42, 1.59)	0.555
Female	905	18	1.00	
Age (years)	2184	600	0.99 (0.96, 1.01)	0.347
Age category				
< 5 years	439	3	0.69 (0.17, 2.74)	0.594
5-11 years	948	12	1.22 (0.48, 3.11)	0.674
12+ years	797	37	1.00	
WAZ score[1]	433	3	2.47 (0.80, 7.60)	0.115
Nutritional status[1]				
Underweight	43	0	NE	
Normal	390	3	1.00	
G6PD status				
Deficient	141	20	2.39 (1.21, 4.75)	0.012
Normal	2038	32	1.00	
Log10 parasitaemia	1606	51	1.12 (0.95, 1.33)	0.191
Hyperparasitaemia				
parasitaemia<100,000 /µL	1479	44	1.00	
parasitaemia>100,000 / µL	135	7	4.65 (1.68, 12.92)	0.003
Haemoglobin (g/dL)	2177	52	1.07 (0.90-1.27)	0.436
No anaemia	1865	46	1.00	
Moderate/severe anaemia[2]	312	6	1.29 (0.50, 3.32)	0.599
Temperature	1602	51	1.24 (0.93, 1.66)	0.143
Fever[3]				
Yes	336	30	1.60 (0.73, 3.53)	0.24

No	1266	21	1.00	
Transmission intensity[4]				
Low	1171	39	1.00	
Moderate	347	12	1.02 (0.03, 36.56)	0.989
High	666	1	0.06 (0.00, 3.88)	0.183
PQ administration				
Yes	1507	39	2.39 (1.22, 4.67)	0.011
No	677	13	1.00	
PQ dose (0.1mg/kg)	2184	52	1.36 (1.08, 1.72)	0.009
Target dose (mg/kg)				
0	677	13	1.00	
Low	326	0	NE	
0.25	424	33	2.48 (1.27, 4.85)	0.008
High	757	6	1.84 (0.52, 6.54)	0.347
Time of PQ				
no PQ	680	13	1.00	
day 0	410	38	2.95 (1.45, 6.01)	0.003
day 2	1104	1	0.18 (0.02, 1.98)	0.162
ACT[5]				
AL	1205	39	2.36 (0.57, 9.80)	0.238
ASAQ	90	9	3.33 (0.80, 13.90)	0.099
DP	889	4	1.00	

[1] evaluated in children < 5years of age, underweight is defined as waz score<-2; [2]includes 1 patients with severe (<7g/dL) anaemia [3]defined as temperature>37.5C or history of fever; [4] Transmission Intensity areas defined based on estimates of P. falciparum prevalence rate (PfPR) [Gething 2011], assuming low transmission for study sites with a PfPR <0.15, moderate transmission if PfPR 0.15 to <0.40 and high transmission if PfPR ≥0.40; ; [5] AL =artemether-lumefnantrine, AP =artemisinin-piperaquine , ASAQ= artesunate-amodiaquine , ASSP= artesunate-sulfadoxine-pyrimethamine, DP=dihydroartemisinin-piperaquine.

Supplementary Table 12. Multivariable model of absolute change in haemoglobin on Day 7 evaluated in studies from sub-Saharan Africa.

Parameter	Absolute change			
	**N[1]**	**change (g/dL)**	**95% CI**	**P-value**
Age/Sex category				
< 5 years	716	-0.784	-0.945, -0.622	<0.001
5-11 years	1,411	-0.517	-0.646, -0.388	<0.001
12+ years females	420	-0.580	-0.725, -0.434	<0.001
12+ males	720	0.000		
Transmission intensity [2]				
Low	1,836	-0.676	-0.853, -0.499	<0.001
Moderate	769	-0.177	-0.348, -0.005	0.043
High	662	0.000		
Pf Malaria Status				
Yes	1,860	-0.624	-0.939, -0.309	<0.001
No or unknown[3]	1,407	0.000		
Log 10 parasitaemia	3,267	***Supplementary Figure 4***		<0.001
Haemoglobin (g/dL)	3,267	-0.404	-0.430, -0.379	<0.001
G6PD status				
Normal	2,670	0.000		
Deficient	193	0.021	-0.231, 0.273	0.871
Not tested	404	-0.040	-0.280, 0.199	0.742
PQ dose (0.1 mg/kg)[4]				
Normal G6PD status	1,992	-0.008	-0.026, 0.010	0.376
G6PD deficient	127	-0.261	-0.336, -0.186	<0.001
G6PD status unknown	333	-0.012	-0.055, 0.032	0.602

[1] N= number of observations in that category; [2] Transmission Intensity defined based on estimates of P. falciparum prevalence rate (PfPR) [Gething 2011], assuming low transmission for study sites with a PfPR <0.15, moderate transmission if PfPR 0.15 to <0.40 and high transmission if PfPR ≥0.40; [3] includes 793 individuals with no malaria and 626 asymptomatic individuals from community who were not tested; [4] N and n, defined as before but refer to individuals who received primaquine in that category;

Supplementary Figure 1. Map of study sites

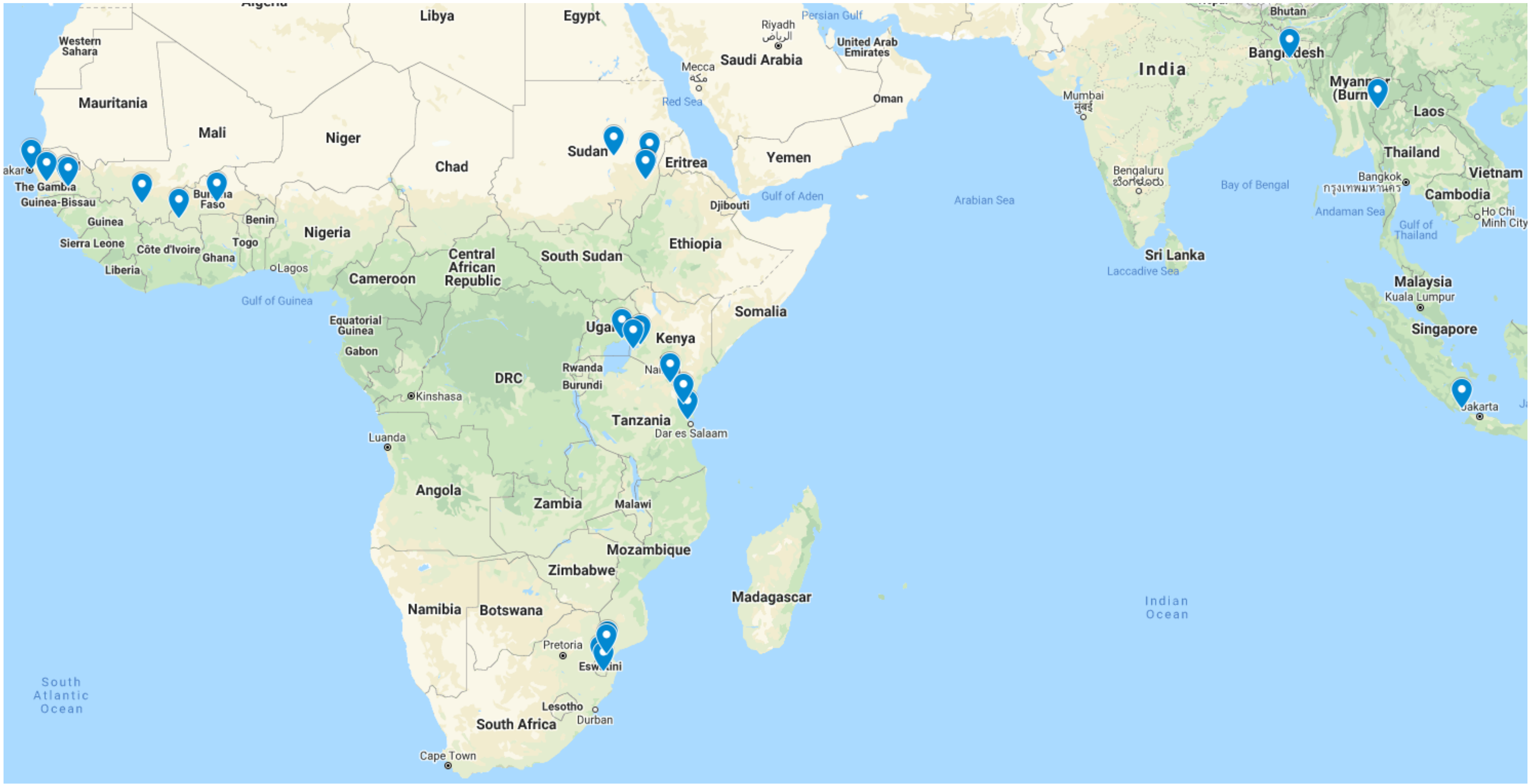

Supplementary Figure 2. Haemoglobin concentrations in G6PD deficient patients not included in the haematology analysis dataset.

Panel A show data from five patients treated with 0.75mg/kg primaquine on day 2 (study ID 18), panel B shows data from 158 patients treated with primaquine 0.25mg/kg on day 1 (study ID 20). Green lines show timing of primaquine administration.

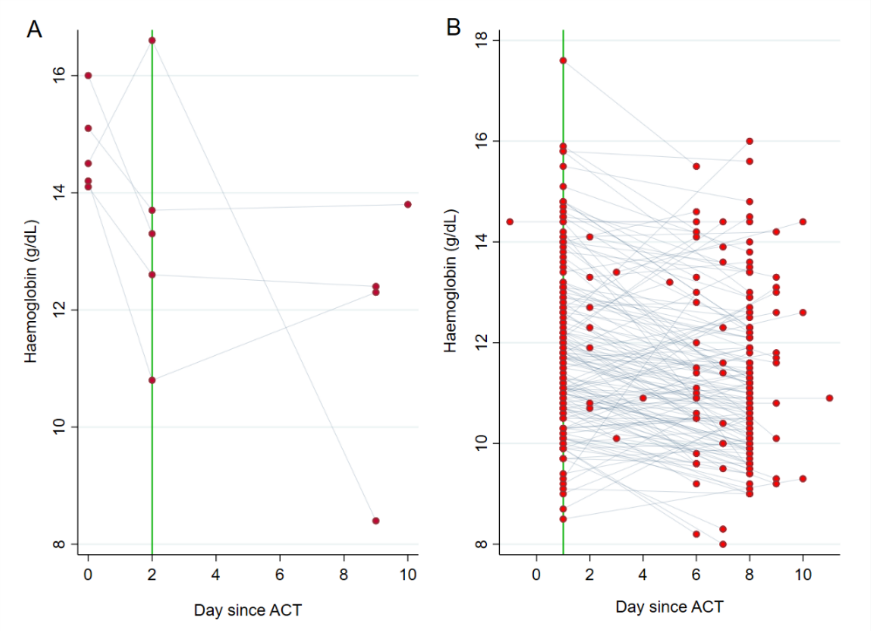

Panel B: Changes in haemoglobin concentration between day 6-8 (one measurement per participant only available and day 1 (when primaquine was given) are smaller than changes between Day 7 and day 0 in other studies with primaquine dose of 0.25mg/kg, by 0.48 g/dL (95%CI 0.12-0.85), p= 0.010. Proportion of participants with moderate anaemia on days 6-8 was also similar to other studies, OR = 0.61 (95%CI 0.21-1.75), p=0.357 and none of the participants reached the severe anaemia threshold of 7g/dL during follow-up. The estimates are adjusted for covariates (using the same fractional polynomials where relevant) as in the final AC7 model, presented in Table 2.

Supplementary Figure 3. Relationship between haemoglobin at baseline and Day 7 as absolute change in patients (A) without G6PD deficiency and (B) with G6PD deficiency.

The dashed orange line represents a fractional fall of 25%. Data points are colour-coded within the PQ target dose administered.

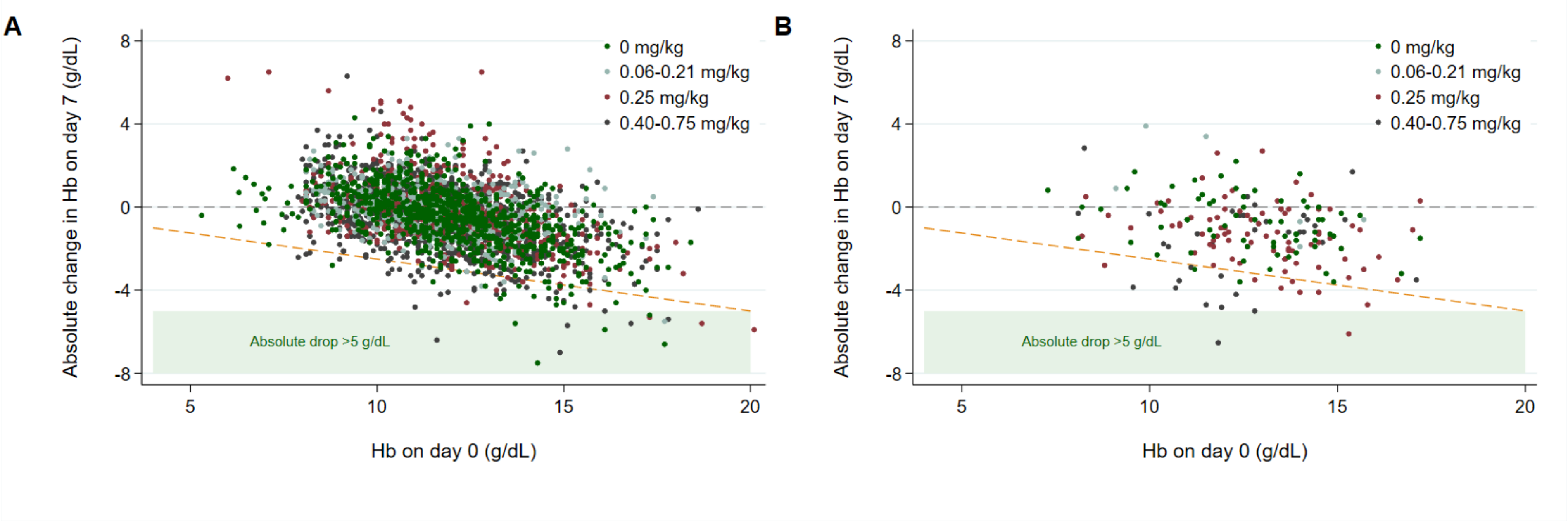

Supplementary Figure 4. Relationship between haemoglobin at baseline and Day 7 as fractional change in patients (A) without G6PD deficiency and (B) with G6PD deficiency.

The dashed orange line represents a fractional fall of 25%. Data points are colour-coded within the PQ target dose administered.

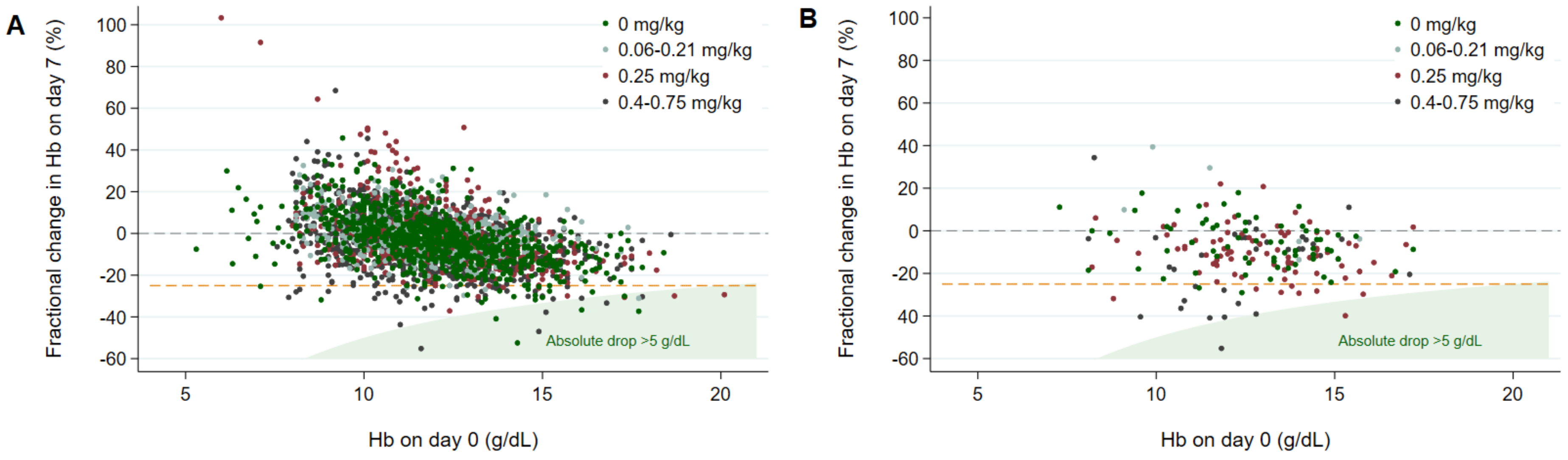

Supplementary Figure 5. Relationship between baseline parasitaemia and haematological endpoints.

A: an absolute change in haemoglobin on Day 7 evaluated in all patients; B: risk of moderate/severe anaemia on Day 7; C: absolute change in haemoglobin on Day 7 evaluated in patients from sub-Saharan Africa

This relationship is adjusted for all independent predictors (shown in Table 2) and evaluated for a child < 5years of age, from low transmission intensity area, normal G6PD status and baseline haemoglobin =12g/dL.

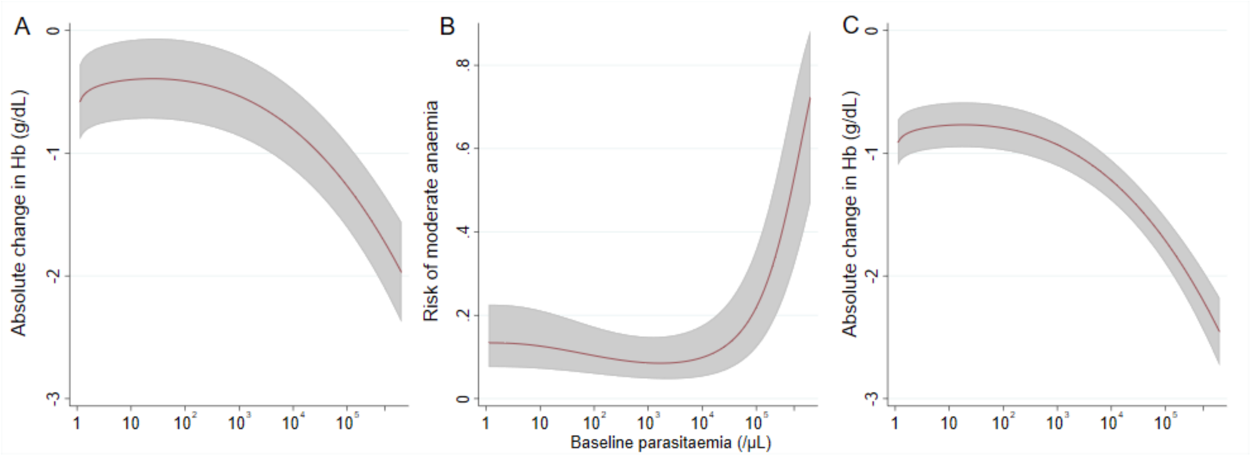

Supplementary Figure 6. Dose-response relationship for haematological endpoints.

Estimated for a child < 5 years of age with G6PD deficiency, from low transmission intensity and parasitaemia of 10,000/µL shown for different baseline haemoglobin.

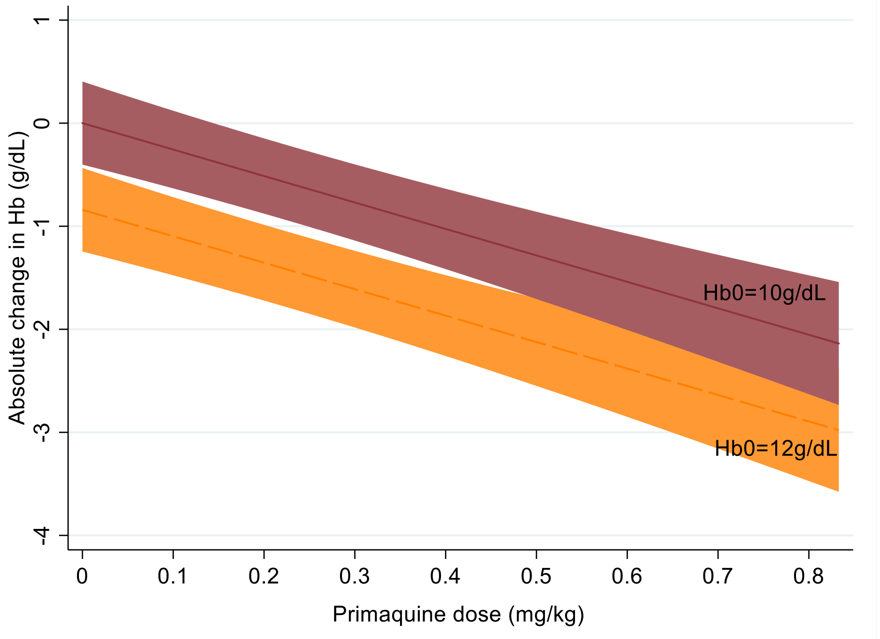

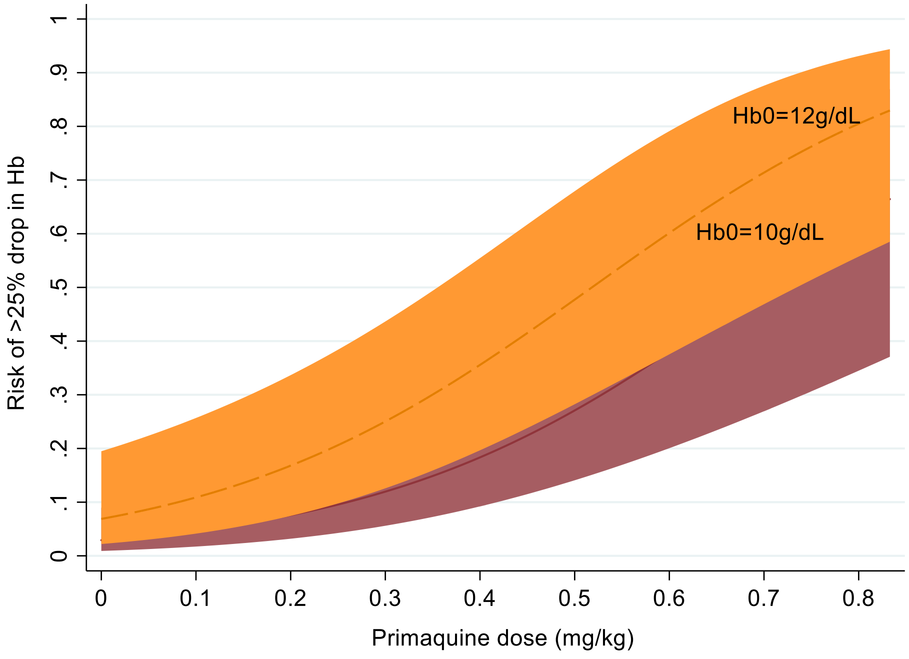

Supplementary Figure 7. Time to recovery by day when anaemia was first recorded.

Time to recovery is measured from the day of PQ administration if anaemia was present at that time, even for participants who already presented with anaemia on previous days (for example on enrolment).

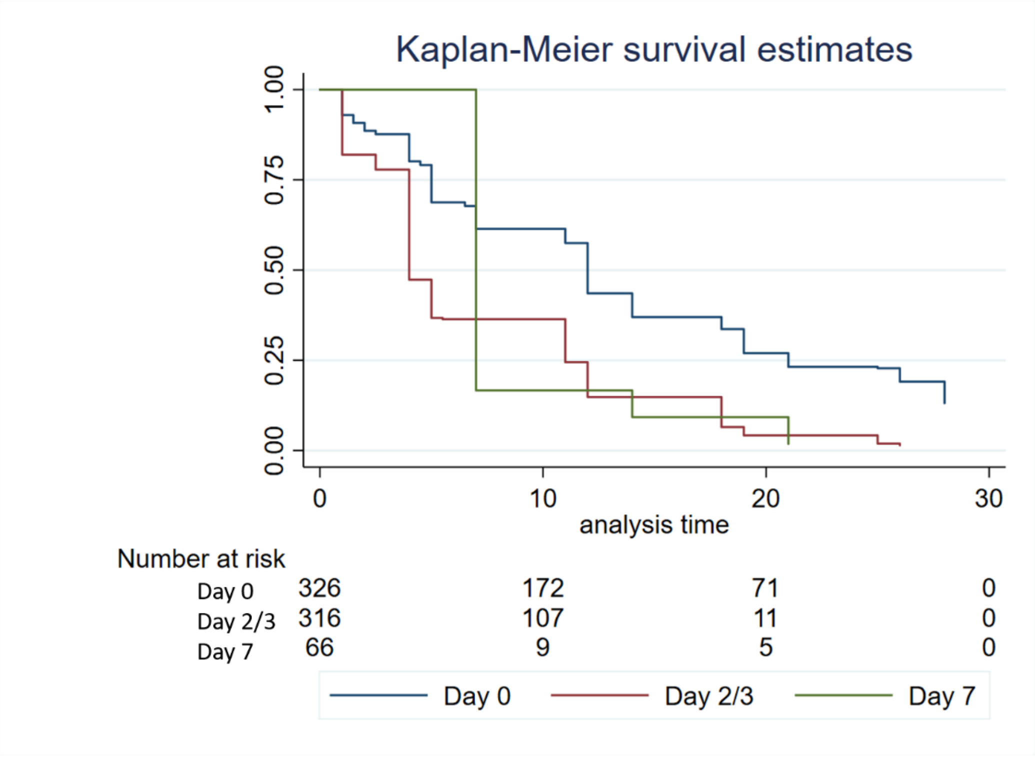

Supplementary Figure 8. Meta-analysis of odds of haemoglobinuria after PQ administration.

OR>1 denotes increased risk of haemoglobinuria in PQ arm. Three studies were excluded from this analysis as there were no haemoglobinuria events recorded

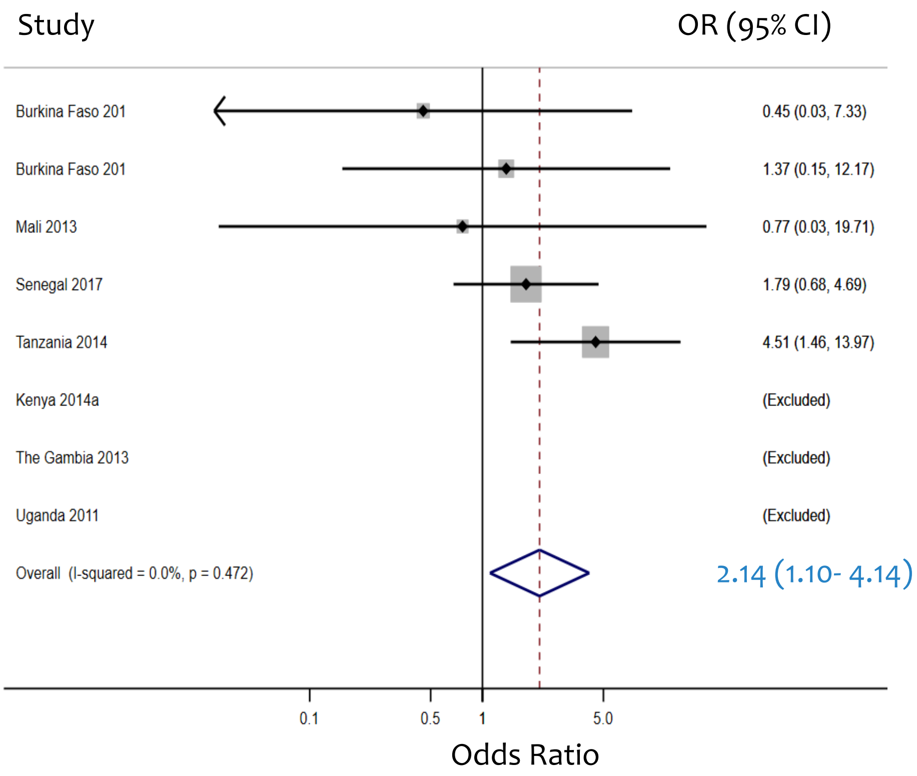

Supplementary Figure 9. Distribution of parasitaemia (observed) used in the simulation study.

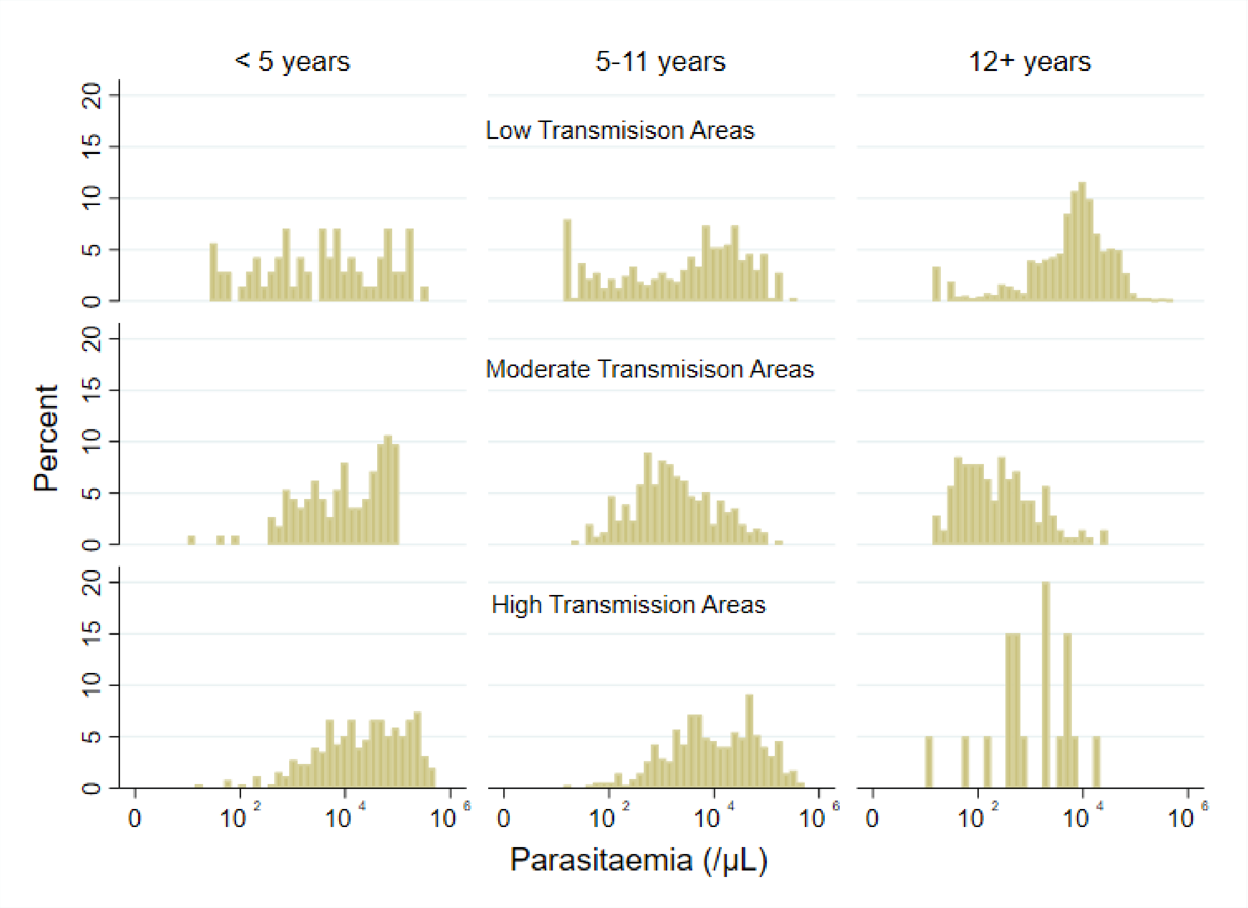

Supplementary Figure 10. Predicted risk of severe anaemia on Day 7 (Hb<7g/dL) in Plasmodium falciparum patients with G6PD deficiency in sub-Saharan Africa.

Risk of Hb<7g/dL is shown for patients treated with ACT (blue line), ACT + 0.25mg/kg primaquine dose (red line) or ACT+0.40mg/kg primaquine dose (green line). Panel A shows results for children <5 years of age, panel B for children 5-11 years of age and panel C for participants 12 years of age or older. Results come from a simulation study from AC7 model presented in Supplementary Table 5.

*prevalence of severe anaemia < 1/ 100,000 simulated participants

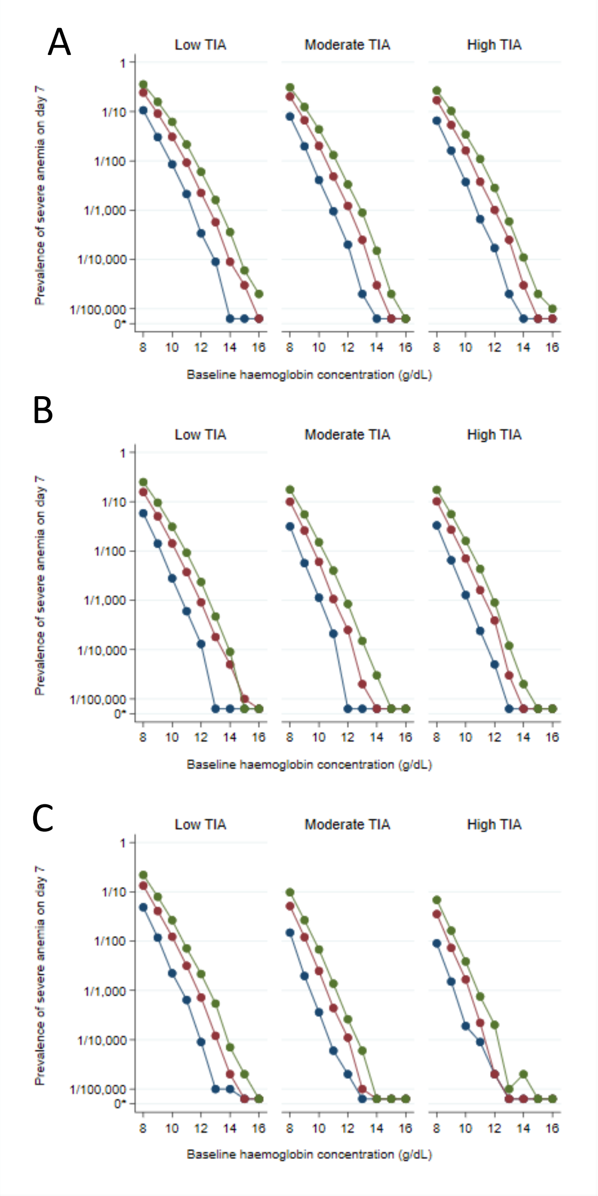

Supplementary Figure 11. Predicted risk of very severe anaemia (Hb<5g/dL) on Day 7 in Plasmodium falciparum patients with G6PD deficiency in sub-Saharan Africa.

Risk of Hb<5g/dL is shown for patients treated with ACT (blue line), ACT + 0.25mg/kg primaquine dose (red line) or ACT+0.40mg/kg primaquine dose (green line). Panel A shows results for children <5 years of age, panel B for children 4-11 years of age and panel C for participants 12 years of age or older. Results come from a simulation study from AC7 model presented in Supplementary Table 5.

*prevalence of severe anaemia < 1/ 100,000 simulated participants

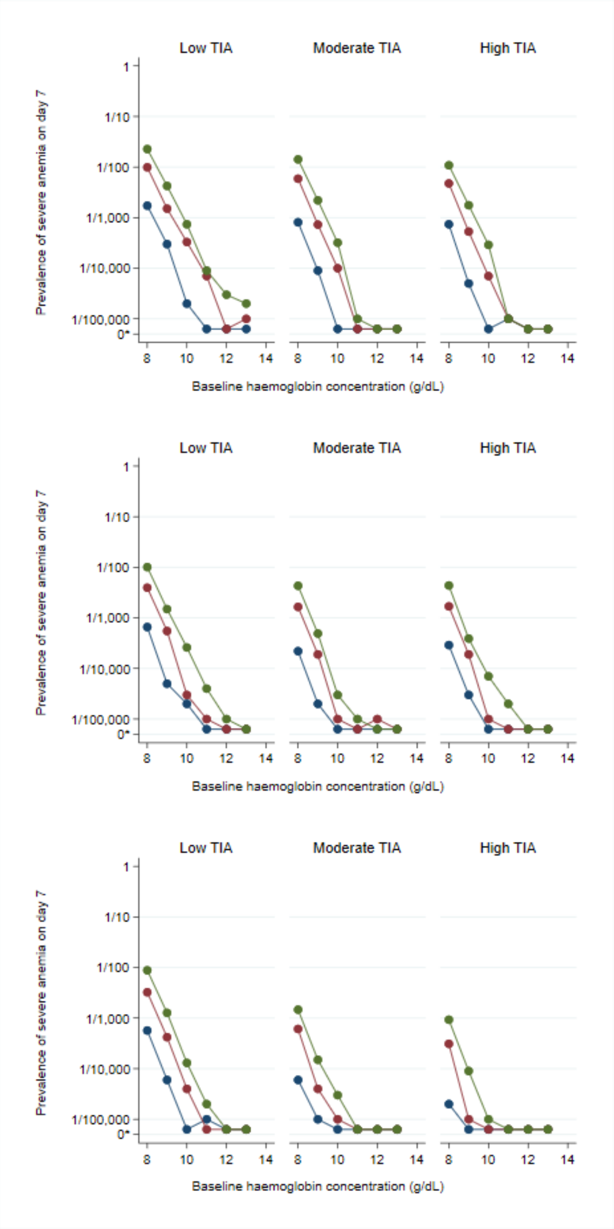

Supplement: Supplementary file 3 — Additional file 3: Table S2. Baseline characteristics of patients in the analysis of haematological response. Table S3. Association between haemoglobin change and baseline value. Table S4. Bi-variable analysis of factors associated with drop in haemoglobin on Day 7. Table S5. Bi-variable analysis of factors associated with anaemia on Day 7. Table S6. Univariable analysis of recovery from anaemia. Table S7. Haemoglobin measurements in patients with severe anaemia at any time. Table S8. Baseline characteristics of patients included in the analysis of adverse events. Table S9. Listing of Serious Adverse Events reported in any study. Table S10. Baseline characteristics of patients included in the analysis of haemoglobinuria. Table S11. Univariable analysis for haemoglobinuria. Table S12. Multivariable model of change in haemoglobin on Day 7 in sub-Saharan Africa. Fig. S1. Map of study sites. Fig. S2. Haemoglobin in G6PD deficient patients not included in the Day 7 analysis. Fig. S3. Relationship between haemoglobin at baseline and Day 7 as absolute change. Fig. S4. Relationship between haemoglobin at baseline and Day 7 as fractional change Fig. S5. Relationship between baseline parasitaemia and haematological endpoints. Fig. S6. Dose response relationship for haematological endpoints. Fig. S7. Time to recovery by day when anaemia was first recorded. Fig. S8. Meta-analysis of odds of haemoglobinuria after PQ administration. Fig. S9. Distribution of parasitaemia (observed) used in the simulation study. Figure S10- Predicted risk of severe anaemia (Hb<7g/dL) on Day 7. Fig. S11. Predicted risk of very severe anaemia (Hb<5g/dL) on Day 7. [file 12916_2022_2504_MOESM3_ESM.pdf]
