# Supplementary material for: Safety of single-dose primaquine as a Plasmodium falciparum gametocytocide: a systematic review and meta-analysis of individual patient data
Source: BMC Med. 2022 Sep 16;20:350. doi: 10.1186/s12916-022-02504-z (PMC9479278; doi:10.1186/s12916-022-02504-z)
Supplement: Supplementary file 4 — Additional file 4: Table S13. Risk of bias assessment. Table S14. Individual participant data not available. Fig. S12. Sensitivity analysis for the main haematological results. [file 12916_2022_2504_MOESM4_ESM.pdf]

# Risk of bias assessment

Supplementary Table 13. Risk of bias assessment ........................................................................ 2
Supplementary Table 14. Individual participant data not available ........................................................... 4
Supplementary Figure 12. Sensitivity analysis for the main haematological results .................................. 5

## Supplementary Table 13. Risk of bias assessment

| Study ID | Study design[1] | Random sequence generation (selection bias) | Allocation concealment (selection bias) | Blinding of participants, personnel (performance bias) Hb Day 7, AEs | Blinding of outcomes assessment (detection bias) Hb Day 7, AEs | Incomplete outcome data (attrition bias) Hb Day 7 | | Other bias AE elicitation method |
|---|---|---|---|---|---|---|---|---|
| | | | | **Primary outcome Hb D7** | **Primary outcome Hb D7** | **n** | **%** | |
| 1 | MDA | Not applicable | Not applicable | Open label | Open label | 295 | 78% | Open questions |
| 2 | RCT | Computer generated | Sealed, opaque envelopes | Open label | Open label, not clear if Hb staff blinded | 692 | 89% | Open questions, direct for dark/'coke' urine |
| 3 | RCT | Computer generated | Sealed, opaque envelopes | Staff unblinded, participants blinded | Open label | 220 | 97% | Checklist questions, incl. for dark urine |
| 4 | RCT | Envelope draw | Sealed, opaque envelopes | Open label | Not reported | 231 | 71% | Checklist |
| 5 | RCT | Envelope draw | Sealed, opaque envelopes | Only pharmacist, unblinded | Blinded | 357 | 97% | Open, some checklist items, incl. dark urine |
| 6 | RCT | Computer generated | Sealed, opaque envelopes | Open label | Open label | 86 | 92% | Not reported |
| 7 | RCT | Computer generated | Sealed, opaque envelopes | Open label | Open label | 50 | 92% | Checklist questions, Hillman for dark urine |
| 8 | RCT | Computer generated | Sealed, opaque envelopes | Only pharmacist, unblinded | Blinded | 461 | 100% | Checklist questions, incl. dark urine |
| 9 | RCT | Envelope draw | Sealed, opaque envelopes | Open label | Open label | 78 | 88% | Question dark urine |
| 10 | RCT | Computer generated | Sealed, opaque envelopes | Open label | Open label | 61 | 69% | Not reported |
| 11 | RCT | Computer generated | Sealed, opaque envelopes | Open label | Hb lab staff blinded, clinical staff unblinded | 267 | 89% | Not reported except Hillman for dark urine |
| 12 | Cohort | Not applicable | Not applicable | Open label | Open label | 94 | 66% | Open questions, Hillmen for dark urine |
| 13 | RCT | Computer generated | Sealed, opaque envelopes | Open label | Hb lab staff blinded, clinical staff unblinded | 103 | 98% | Checklist questions, incl. for dark urine |
| 14 | MDA | Not applicable | Not applicable | Open label | Open label | 843 | 70% | Not reported |
| 15 | RCT | Computer generated | Sealed, opaque envelopes | Open label | Open label | 373 | 98% | Checklist |
| 16 | RCT | Computer generated | Sealed, opaque envelopes | Open label | Open label | 139 | 60% | Open and some checklist items |
| 17 | RCT | Computer generated | Sealed, opaque envelopes | Only pharmacist, unblinded | Blinded | 114 | 100% | Not reported |

| | | | | | | | | |
|---|---|---|---|---|---|---|---|---|
| 18 | Cohort | Not applicable | Not applicable | Open label | Open label | | | Checklist questions, incl. for dark urine |
| 19 | RCT | Computer generated | Sealed, opaque envelopes | Staff blinded, participants unblinded on request by non-investigator | Blinded | 81 | 95% | Open and some checklist questions |
| 20 | MDA | Not applicable | Not applicable | Open label | Open label | | | Not reported |

[1]MDA = mass drug administration, RCT = randomised controlled trial; D7 = Day 7

## Supplementary Table 14. Individual participant data not available

| Author, date | PubMed ID | Study Design | Location | Year | ACT | Follow up (days) | Age | G6PD exclusion | Target primaquine dose (mg/kg) | N | Summary of haemoglobin outcome | Summary of adverse events |
|---|---|---|---|---|---|---|---|---|---|---|---|---|
| Arroyo-Arroyo, 2( | 28749756 | 3 sequential dose groups ACT plus primaquine | Colombia | 2010-2014 | AL | 7 | All | Not tested | 1) 0.75 day 3, 2) 0.50 day 1 and 0.25 day 3, 3) 0.25 days 1, 2 and 3 | 60 | Not applicable | No volunteers had adverse events possibly associated with the antimalarial treatment. The signs and symptoms observed in all patients were indistinguishable from the common malaria symptoms |
| Lin,2017* | 28591198 | Randomised controlled trial, ACT with/without primaquine | Cambodia | 2012-2014 | DP | 42 | 18-65 | Severe | 0.75 day 3 | 101 | Among 8/101 with mild or moderate G6PD deficiency, a greater drop in haemoglobin at day 7 was seen with primaquine but not statistically significant: median fractional reduction in haemoglobin 24.8% (range 0.9% to 29.8%) in 6 given primaquine versus 7.1% (-4.4% and 18.7%) in 2 not given primaquine (p = 0.14). The largest drop was 9.9 g/dL from a baseline of 14.1; all Hgb values were >11.0 g/dL by day 14. In G6PD normal subjects, haemoglobin was only measured to day 3, at which time there was no change in the fractional drop between arms (9.8% versus 9.3% in the primaquine and non-primaquine groups, respectively, p = 0.79). | |
| Shah, 2013* | 23587943 | Meta-analysis of sites with / without primaquine | India | 2009-2010 | ASSP | 28 | > 1 year | Not tested | 0.75 day 2 | 1372 | Not applicable | Adverse events reported included vomiting (8) and fever (2) in the AS+SP-alone arm and vomiting (14) and jaundice (1) in the primaquine arm. Only 5/14 patients who experienced vomiting did so on the day of primaquine administration. The jaundice occurred in a 5-year-old on day 14 and self-resolved |
| Smithius 1010 | 20832366 | Randomised controlled trial, ACT with/without primaquine | Myanmar | 2008-2009 | AM, ASAQ, DP, AL | 63 | > 1 year | Not tested | 0.75 day | 808** | No day 7 data | 599 (74%) reported adverse events. 13 (1.6%) vomited within the first hour, none after treatment. The ony side-effect attributed to primaquine was abdominal pain. |
| Tun, 2016 | 27036739 | Single arm | Myanmar | 2013-2014 | DP | 42 | 6 months to 65 years | Not tested | 0.25mg/kg day 0 | 116 | There were no clinically significant falls in haemoglobin, or haemoglobinuria | Study medications generally well tolerated although 6 vomited day 0 medication, and 1 vomited on day 2. One serious adverse event, a death through road traffic accident 2 weeks after the last visit |

AL: artemether-lumefantrine, ASAQ:artesunate amodiaquine, AM:artesuate-mefloquine, ASSP:artesunate sulphadoxine-pyrimthamine, DP:dihydroartemisinin piperaquine,

*Unclear if requested

**Included mixed infections

**Supplementary Figure 12. Sensitivity analysis for the main haematological results**

Figures show estimates and their 95% CI for the effect of primaquine dose ( in G6PD deficient subjects in the left-hand-side panels and in G6PD normal subjects in the right-hand panels)  from the final multivariable model (in blue ) and in the same model re- fitted after exclusion of each study in turn (in green). Two endpoints are presented: absolute change in haemoglobin on Day 7 (upper panels) and risk of moderate anaemia on Day 7 (lower panels). Red line show the effect estimated in the final model.

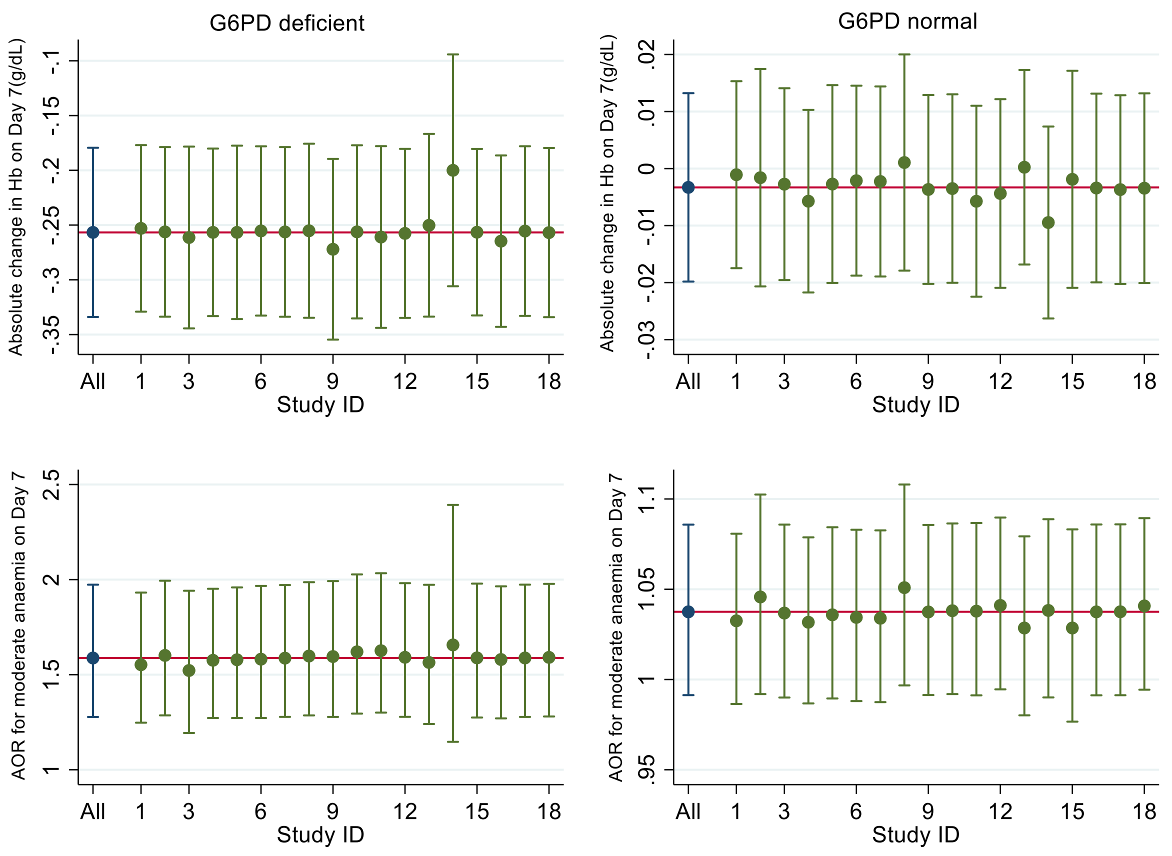


Coefficient of variation for the estimates was (from top left, clockwise) 5.7%, 75.8%, 3.9% and 14.5%.

Large variability for the second model was due to the estimates being very close to 0, nonetheless the results were very consistent throughout.
